# DNA Topoisomerases Participate in Fragility of the Oncogene *RET*

**DOI:** 10.1371/journal.pone.0075741

**Published:** 2013-09-11

**Authors:** Laura W. Dillon, Levi C. T. Pierce, Christine E. Lehman, Yuri E. Nikiforov, Yuh-Hwa Wang

**Affiliations:** 1 Department of Biochemistry, Wake Forest School of Medicine, Winston-Salem, North Carolina, United States of America; 2 Department of Chemistry and Biochemistry, University of California-San Diego, La Jolla, California, United States of America; 3 Department of Cancer Biology, Wake Forest School of Medicine, Winston-Salem, North Carolina, United States of America; 4 Department of Pathology and Laboratory Medicine, University of Pittsburgh, Pennsylvania, United States of America; University of Oklahoma, United States of America

## Abstract

Fragile site breakage was previously shown to result in rearrangement of the *RET* oncogene, resembling the rearrangements found in thyroid cancer. Common fragile sites are specific regions of the genome with a high susceptibility to DNA breakage under conditions that partially inhibit DNA replication, and often coincide with genes deleted, amplified, or rearranged in cancer. While a substantial amount of work has been performed investigating DNA repair and cell cycle checkpoint proteins vital for maintaining stability at fragile sites, little is known about the initial events leading to DNA breakage at these sites. The purpose of this study was to investigate these initial events through the detection of aphidicolin (APH)-induced DNA breakage within the *RET* oncogene, in which 144 APH-induced DNA breakpoints were mapped on the nucleotide level in human thyroid cells within intron 11 of *RET*, the breakpoint cluster region found in patients. These breakpoints were located at or near DNA topoisomerase I and/or II predicted cleavage sites, as well as at DNA secondary structural features recognized and preferentially cleaved by DNA topoisomerases I and II. Co-treatment of thyroid cells with APH and the topoisomerase catalytic inhibitors, betulinic acid and merbarone, significantly decreased APH-induced fragile site breakage within *RET* intron 11 and within the common fragile site FRA3B. These data demonstrate that DNA topoisomerases I and II are involved in initiating APH-induced common fragile site breakage at *RET*, and may engage the recognition of DNA secondary structures formed during perturbed DNA replication.

## Introduction

The oncogene *RET* is involved in recurrent chromosomal rearrangements found in thyroid and more recently in lung cancer [1–4]. In thyroid cells, it rearranges with various genes in a class of rearrangements known as *RET/PTC* rearrangements, which are known to be carcinogenic for thyroid cells and result in development of papillary thyroid carcinoma (PTC) [5]. The incidence of thyroid cancer has steadily increased over the past several decades; in the United States alone, cases have doubled in the past decade and nearly tripled since the early 1970s [6,7]. Interestingly, the increase in thyroid cancer is almost entirely attributable to an increase in PTC [7]. Approximately 20% of all PTC cases are due to *RET/PTC* rearrangements [5]. The most common form of *RET/PTC* rearrangement is the *RET/PTC1* type, where *RET* translocates with *CCDC6* [8]. *RET* and *CCDC6* are both located within common chromosomal fragile sites, FRA10G and FRA10C, respectively. Recently we found that the formation of *RET/PTC1* rearrangements can be induced in human thyroid cells through treatment with fragile site-inducing chemicals [9]. Therefore, it is conceivable that exposure to chemicals that can induce fragile sites may contribute to the increasing rates of thyroid cancer.

Chromosomal fragile sites are specific regions of the genome that exhibit gaps or breaks on metaphase chromosomes under conditions that partially inhibit DNA replication [10]. These sites often co-localize with regions deleted, amplified, or rearranged in cancer [11]. Over half of all known simple recurrent chromosomal translocations in cancer have breakpoints located within at least one fragile site [12]. Mutational signatures of some unexplained homozygous deletions in cancer cell lines match those found in fragile site regions [13]. Furthermore, fragile site-inducing conditions introduced *in vivo* deletions within the tumor suppressor gene *FHIT* and generated oncogenic *RET/PTC1* rearrangements similar to those in patients [9,14].

Although a strong connection between fragile sites and cancer has been established, little is known about the initial events leading to DNA breakage at these sites. Chromosomal fragile sites are traditionally defined cytogenetically as unstained gaps with an average size of 3 Mb. Some common fragile sites have been defined on the molecular level, where DNA breakage is observed over large regions up to several megabases in size [15]. Unlike rare fragile sites, which consist of repeated sequence elements present in less than 5% of the population and inherited in a Mendelian manner [16], common fragile sites are present in all individuals and have no known consensus sequence [17]. Common fragile sites are further characterized based on the culture conditions known to induce breakage within these regions, the most common being aphidicolin (APH), an inhibitor of DNA polymerases α, β, and δ [18,19]. Although no consensus sequence is known for common fragile sites, several characteristics are shared among many sites studied to date, including being late-replicating [20–23], located within large genes [10], containing highly flexible AT-rich sequences [24,25], and having the potential to form highly stable DNA secondary structures [25–27]. Recently, in studying of the human chromosome 10 sequence, we found that APH-induced common fragile sites are predicted to form more stable DNA secondary structures that cluster with greater density than non-fragile regions [28]. One proposed mechanism for common fragile site breakage is that replication stress results in a long stretch of single-stranded DNA and subsequent formation of stable DNA secondary structures, which can pause polymerase progression, resulting in incomplete replication at fragile sites and ultimately DNA breakage [10]. In addition to DNA replication, transcription of large genes at fragile sites can result in the formation of stable R-loop structures that ultimately result in common fragile site breakage [29]. Triplet repeat expansions, including those observed at rare fragile sites, also form stable R-loops during transcription, most likely influenced by the formation of stable DNA secondary structures on the non-template strand [30–32].

DNA topoisomerases play a critical role in maintaining chromosome structural integrity during DNA processes such as replication or transcription by regulating DNA supercoiling and removing knots in the genomic material [33]. During replication and transcription, topoisomerase I alleviates DNA supercoiling by transiently inducing a single-strand DNA break and then re-ligating at the cleavage site. Topoisomerase IIα modulates DNA supercoiling and removes knots and tangles in the DNA formed during replication by transiently inducing a double-strand DNA break and then re-ligating at the cleavage site. Additionally, DNA topoisomerases I and II can recognize and preferentially cleave DNA at regions capable of forming stable DNA secondary structures [34–36], similar to those predicted or formed at fragile sites. Furthermore, normal topoisomerase I activity is vital for common fragile site breakage [37,38].

The critical role of DNA topoisomerases in replication and transcription, their recognition of DNA secondary structures, and the involvement of topoisomerase I in fragile site breakage, prompted us to directly investigate the role of these enzymes in initiating common fragile site breakage. The nucleotide locations of APH-induced DNA breaks within intron 11 of the *RET* oncogene, the major breakpoint cluster region in patients with PTC [39], were determined using ligation-mediated PCR (LM-PCR) and were at or near topoisomerase I and/or II predicted DNA cleavage sites. Furthermore, using DNA secondary structure predictions of the intron 11 sequence, the APH-induced breakpoints were present in structural features known to be recognized by topoisomerases I and II. Finally, treatment of thyroid cells with low doses of topoisomerase catalytic inhibitors significantly reduced the rate of APH-induced DNA breakage within intron 11 of *RET*, as well as intron 4 of *FHIT*, located within the most active common fragile site FRA3B, to levels observed in untreated samples. These results support the involvement of DNA topoisomerases I and II in the initiation of DNA breakage at APH-induced common fragile sites, possibly through recognition of DNA secondary structures formed during perturbed DNA replication.

## Materials and Methods

### Cell line and culture conditions

Experiments were performed on HTori-3 cells, a human thyroid epithelial cell line transfected with an origin-defective SV40 genome. They are characterized as immortalized, partially transformed, differentiated cells having three copies of chromosome 10 with intact *RET* loci and preserve expression of thyroid differentiation markers such as thyroglobulin production and sodium iodide symporter [40]. They also contain one copy of chromosome 3, and four copies of chromosome 12. The cells were purchased from the European Tissue Culture Collection and grown in RPMI 1640 medium (Invitrogen) supplemented with 10% fetal bovine serum.

### Cell treatments and fragile site induction

For breakpoint detection, HTori-3 cells (1x10^5^) were plated in 6-well plates and treated 18 hours later for 24 hours with 0.4 µM APH, in the presence or absence of 3 µM merbarone, 6 nM betulinic acid (BA), or 150 nM CPT-11 (all from Sigma). For detection of DNA topoisomerase I and II cleavage sites, HTori-3 cells were plated in the same manner, and treated for 1.5 hours with either 10 µM CPT-11 or 10 µM VP-16 (Sigma).

DNA breaks were directly introduced to HTori-3 genomic DNA through digestion with the restriction enzyme BanI or XbaI (New England BioLabs). DNA breaks were induced within intact nuclei isolated from HTori-3 cells [isolation of nuclei was performed as described in [41]] through treatment with BanI, after which the genomic DNA was isolated.

### DNA breakpoint mapping by LM-PCR

DNA breaks were identified within intron 11 of *RET* using four sets of primers (Table S1), two sets detecting breaks on one DNA strand and two sets detecting breaks on the complementary strand, and each set covering a DNA region of approximately one Kb. Each primer set consists of a 5’-biotinlyated primer that extends to the breakpoint, and two nested primers, used separately in the first and second rounds of PCR to amplify the DNA. DNA breaks within FRA3B were isolated using a set of primers corresponding to intron 4 of *FHIT* (Table S1) [9], which is a hotspot of APH-induced DNA breakage in FRA3B [40,42]. The primers: 12p12.3-1, -2 and -3 [9] were used to detect DNA breaks within a non-fragile region, 12p12.3 [25].

Detection of DNA breakpoints following drug treatment was performed as previously described with modifications [9]. Briefly, genomic DNA was isolated from HTori-3 cells with or without treatment. Primer extension was performed using 200 ng of DNA at 45°C with DNA Sequenase (Affymetrix, Inc.), and the DNA breaks were isolated through ligation of the LL3/LP2 linker, and then using streptavidin beads. The ligation conditions known to favor blunt-ended DNA ligation were used [43], with lower ATP concentrations (50 µM) and the addition of hexamminecobalt chloride (1.5 µM). Amplification of these DNA breaks was achieved by nested PCR of the extension-ligation products, using the equivalent of 8 ng of genomic DNA per reaction (for all treatments except APH + CPT-11, where 4 ng was used). The final PCR products were resolved by electrophoresis on a 1.3% agarose gel. Each band observed on the gel corresponds to a break isolated within the 1 kb region of interest. Each experimental replicates includes 6 LM-PCR reactions (48 ng genomic DNA), and breakage frequency is referred as DNA breaks per 100 ng DNA per gene locus. More than three experimental replicates (specified in the figure legends) were performed for each treatment. The starting material for each replicate was obtained from a separate cell treatment. The size of the bands observed was confirmed by DNA sequencing. The nucleotide location of the breakpoints was determined from the sequencing results by identifying the nucleotide adjacent to the LL3/LP2 linker sequence.

### DNA secondary structure prediction by Mfold

The DNA sequence of intron 11 of *RET* was obtained from NCBI (human genome build 37.2, Chr10: 43610185-43612031). Using the Mfold program [44], the potential of single-stranded DNA to form stable secondary structures can be predicted. The secondary structure forming potential of *RET* intron 11 was analyzed by inputting 300-nt segments with 150-nt shift increments into the Mfold program. We choose the 300-nt length because it equals the length of an Okazaki initiation zone of the DNA replication fork in mammalian cells, which possesses a single-stranded property during DNA replication [45,46]. The default [Na+], [Mg_2_+], and temperature used were 1.0 M, 0.0 M, and 37°C, respectively. The most stable predicted DNA secondary structure for each 300-nt segment was used to analyze the location of APH-induced DNA breaks within intron 11 of *RET*.

### Cell survival analysis following drug treatment

To analyze cell viability, HTori-3 cells (1x10^5^) were plated in 6-well plates and treated 18 hours later with 0.4 µM APH in the presence or absence of topoisomerase inhibitors for 24 hours. Cell were harvested by trypsinization, washed with phosphate-buffered saline (PBS, Invitrogen), and re-suspended in PBS containing 2 µg/mL propidium iodide. Cell viability was then determined using a Becton Dickinson FACSCalibur flow cytometer. Titrations of betulinic acid (3 nM to 3 µM), merbarone (1 to 100 µM), and CPT 11 (3 nM to 10 µM) were performed to determine optimal dosages in HTori-3 cells. After determining an optimal range of doses for each topoisomerase inhibitor alone, titrations were also performed for betulinic acid (3 nM to 0.3 µM), merbarone (1 to 10 µM), and CPT 11 (150 to 500 nM) in combination with 0.4 µM APH.

To analyze active apoptosis, HTori-3 cells (1x10^5^) were plated, treated and trypsinized as described above. Cells were then washed with PBS, and re-suspended in 1X Annexin V binding buffer. Annexin V stain (BD Biosciences) was then added to each sample and incubated for 15 minutes. Early apoptotic cells were quantified using a BD Accuri C6 flow cytometer.

HTori-3 cells (1x10^5^) were quantified using a hemocytometer and plated in 6-well plates. Cells were then treated, harvested as above and re-plated in fresh media. Cells were quantified using a hemocytometer while re-plating and after cells had been allowed to recover for an additional 24 hours in chemical-free media.

To determine the distribution of cells in each phase of the cell cycle, HTori-3 cells (2x10^5^) were treated as above, washed with PBS, and re-suspended in 100% cold ethanol, while gently vortexing. Cells were incubated overnight, and then washed and resuspended in PBS containing 50 µg/mL propidium iodide and 100 µg/mL RNase. The cell cycle profile was analyzed using a BD Accuri C6 flow cytometer, and ModFit LT (Verity Software House) was used to determine the percentage of cells in each cell cycle phase.

### Statistical analysis

All data are presented as the mean ± standard derivation (SD) or as a percentage. All statistical analyses were performed using two-tailed Student’s t-test.

## Results

### Identification of APH-Induced DNA Breakpoints Within Intron 11 of RET

To investigate the initial events of fragile site-induced DNA breakage, the nucleotide location of APH-induced DNA breakpoints within intron 11 of *RET* was identified by LM-PCR. The translocation of *RET* with various partner genes is the hallmark of *RET/PTC* rearrangements, and the major breakpoint cluster regions observed in patients occur within intron 11 [39]. Previously, we established using LM-PCR that APH treatment induces DNA breakage within intron 11 of *RET* in the human thyroid epithelial cell line HTori-3, and this breakage is specific to fragile sites [9]. Here, the entire *RET* intron 11 was examined by the same method for APH-induced breakpoints using genomic DNA isolated from HTori-3 cells following treatment with 0.4 µM APH for 24 hours. In short, the genomic DNA was subjected to primer extension using biotinylated primers specific for the region of interest (see Materials and Methods; Table S1).

**Figure 1:**
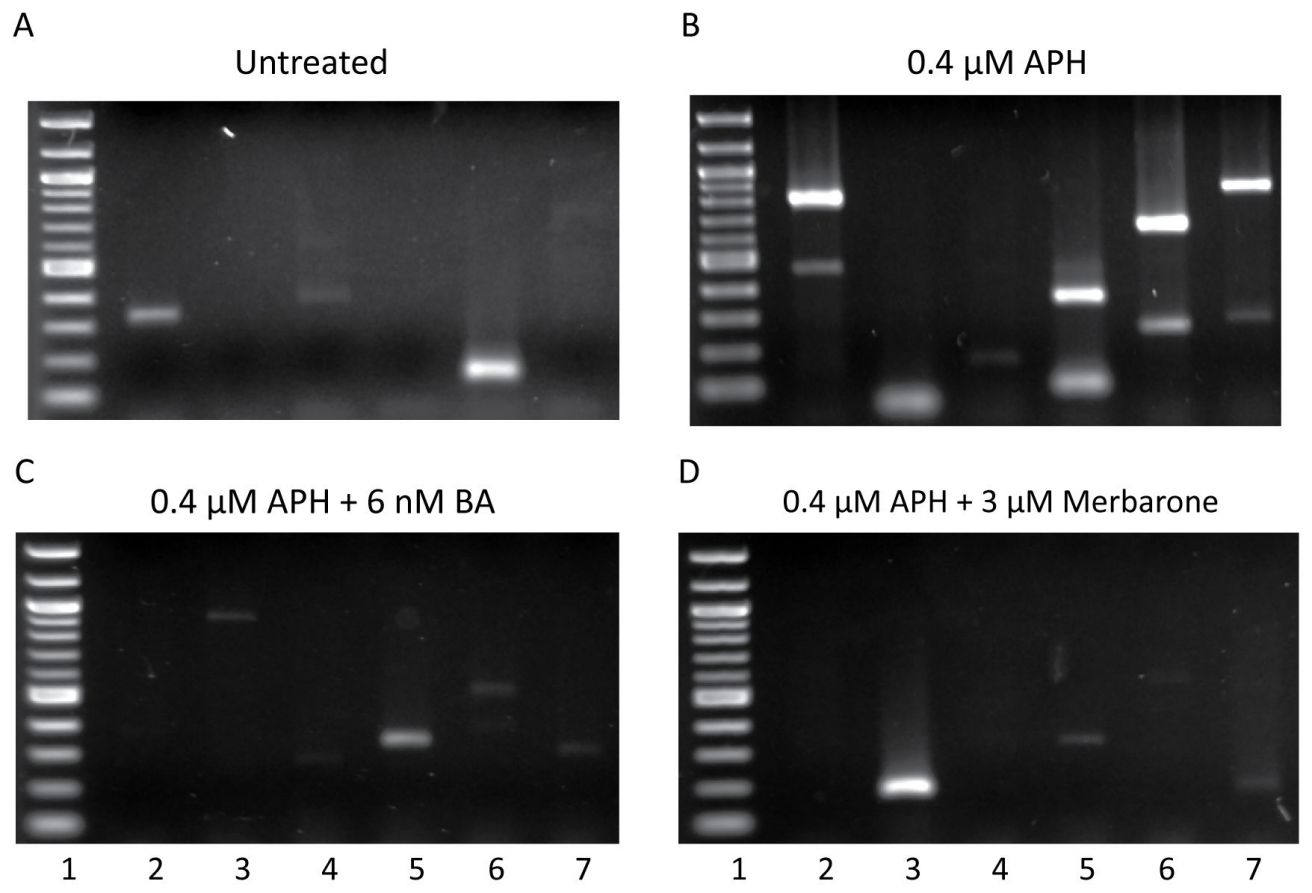
LM-PCR detection of DNA breaks within intron 11 of *RET*. DNA breaks formed in intron 11 of *RET* were detected by LM-PCR without treatment (A) or following 24-hour treatment with 0.4 µM APH alone (B) or in combination with the DNA topoisomerase I and II catalytic inhibitors 6 nM betulinic acid (C) or 3 µM merbarone (D). Representative gels are shown. Each lane represents a separate PCR reaction using DNA from approximately 1300 cells. The first lane of each gel is a 100-bp molecular weight ladder.

Upon reaching a DNA break, the synthesis reaction terminates, resulting in a blunt-ended DNA molecule, which was then captured by linker ligation. The linker-attached DNAs were isolated using streptavidin beads, amplified by two rounds of nested PCR, and visualized by agarose gel electrophoresis (Figure 1). Each lane on the agarose gel represents DNA isolated from approximately 1300 cells and each band on the gel represents a DNA break isolated within *RET* intron 11. A total of 144 DNA breaks were isolated within the 1847-bp intron 11 sequence of *RET* on both DNA strands using four sets of primers, sets 1-4 (Figure 1B; Table S2). DNA breakage within *RET* intron 11 was observed at a frequency of 4.08 ± 1.50 DNA breaks per 100 ng genomic DNA per 1kb locus, significantly more than the rate of DNA breakage without treatment (0.9 ± 0.50 breaks/100 ng DNA/locus, *P* = 2.50E-4, using a two-tailed Student’s t-test; Table 1, Figure 1A).

**Table 1:** Frequency of DNA breakage at *RET* intron 11, FRA3B, and non-fragile 12p12.3 as detected by LM-PCR.

	**DNA Breaks/100 ng DNA/Locus (±SD**)		
**Treatment**	**RET**	**FRA3B**	**12p12.3**
Untreated	0.90 ± 0.50	3.75 ± 0.93	0.13 ± 0.26
0.4 µM APH	4.08 ± 1.50	18.40 ± 2.04	0.13 ± 0.26
0.4 µM APH + 6 nM BA	1.25 ± 0.58	3.75 ± 1.74	-
0.4 µM APH + 3 µM Merbarone	0.97 ± 0.38	5.00 ± 2.80	-
0.4 µM APH + 150 nM CPT11	23.06 ± 3.75	36.98 ± 9.56	1.17 ± 0.50

**Figure 2:**
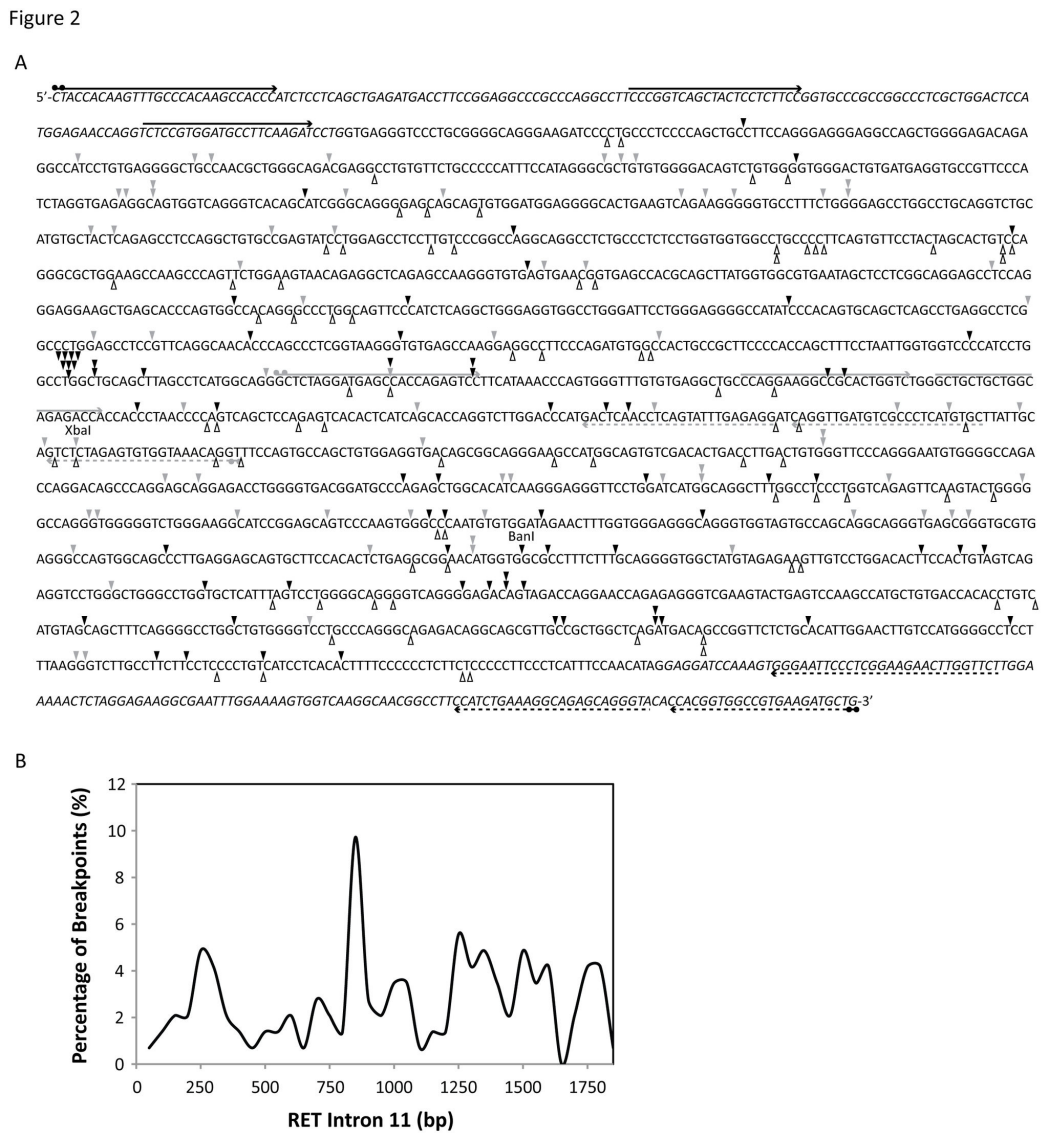
Location of APH-induced DNA breakpoints within intron 11 of *RET* detected by LM-PCR. (A) The location of 144 APH-induced DNA breakpoints isolated within intron 11 of *RET* by LM-PCR were determined by DNA sequencing (arrowheads). DNA breaks identified on the strand shown by the sequence are indicated by black arrowheads, and on the complementary strand by grey arrowheads. Open arrowheads indicate the locations of known patient breakpoints observed in PTC tumors containing *RET/PTC* rearrangements [39,47–50]. The location of BanI and XbaI digestion sites within intron 11 are labeled. *RET* primer sets (see Table S1) are indicated by arrows. Lines with circles are dual biotin-labeled primers followed by two nested primers. The dashed black lines represent primer set 1, dashed grey lines primer set 2, solid black lines primer set 3, and solid grey lines primer set 4. The sequence of intron 11 is displayed along with the flanking exon 10 and 11 sequences, shown in italics. (B) The distribution of APH-induced DNA breakpoints within intron 11 are depicted as a smooth curve fit of the percentage of breakpoints (y axis) located every 50 bp of intron 11 in a 5’ to 3’ direction (x axis).

To assure the location of the APH-induced breakpoints representing the initial events of APH-induced fragile site breakage within this region, we first tested that the LM-PCR procedure could accurately identify the nucleotide location of a DNA break, and that the breaks being detected were not due to premature termination of the primer extension reaction. Genomic DNA isolated from HTori-3 cells was digested with either the restriction enzyme BanI or XbaI. The LM-PCR products from the *RET* primer set 1 (Table S1) were expected to be 454 bp and 864 bp in size, respectively, for BanI and XbaI digested DNAs (Figure 2A). PCR products corresponding to the correct sizes were observed for the digested DNA samples, and DNA sequencing revealed that 100% of the BanI or XbaI-induced breaks corresponded to the correct nucleotide location (data not shown). These results verify that LM-PCR is a valid method for identification of DNA breakpoints up to about 1Kb from the initial biotinylated primer.

Next, we examined whether the locations of DNA breaks being detected by LM-PCR reflect true APH-induced breaks, not the consequences of subsequent repair processes or DNA purification procedures. Intact HTori-3 nuclei were treated with BanI, after which genomic DNA was isolated and analyzed by LM-PCR using *RET* primer set 1. After DNA sequencing of 28 breakpoints generated by the LM-PCR, 79% of breakpoints located to the predicted nucleotide, while the remaining breakpoints contained deletions up to 5 bp, which may be the result of exonuclease digestion (data not shown). Together, these results show that the nucleotide locations of DNA breaks identified by LM-PCR mostly correspond to the initially induced breaks formed inside the cell.

The nucleotide location of the 144 APH-induced DNA breakpoints was determined by sequencing of the PCR products. APH treatment induced DNA breakage throughout intron 11 on both DNA strands (Figure 2A). Interestingly, the breakpoints formed a notable pattern of clusters at approximately every 250 bp, equivalent to spacing between nucleosomes (Figure 2B). Next, the locations of the APH-induced breakpoints were compared with the location of known breakpoints found in PTC tumors containing *RET/PTC* rearrangements (Figure 2A; Figure S1, Table S2) [39,47–50]. We found that 81 (58%) of the APH-induced breakpoints had a known patient breakpoint located within 0 to 20 bp. While the locations of breakpoints identified in PTC tumors were isolated following rearrangement, the APH-induced breakpoints were determined before a translocation event. In most PTC tumors, small insertions or deletions ranging from 1 to 18 bp have been observed surrounding the fusion points.

These results reveal that APH treatment induces DNA breakage throughout intron 11 of *RET*, and that many of these breakpoints are located at or near breakpoints found in tumors from patients. We also show that the LM-PCR can accurately identify the nucleotide location of a DNA breakpoint. Therefore, the locations of these breakpoints can be used to identify initial events in APH-induced fragile site breakage within the *RET* oncogene.

### Location of APH-induced DNA Breakpoints Relative to Predicted Topoisomerase I and IIα Cleavage Sites

**Figure 3:**
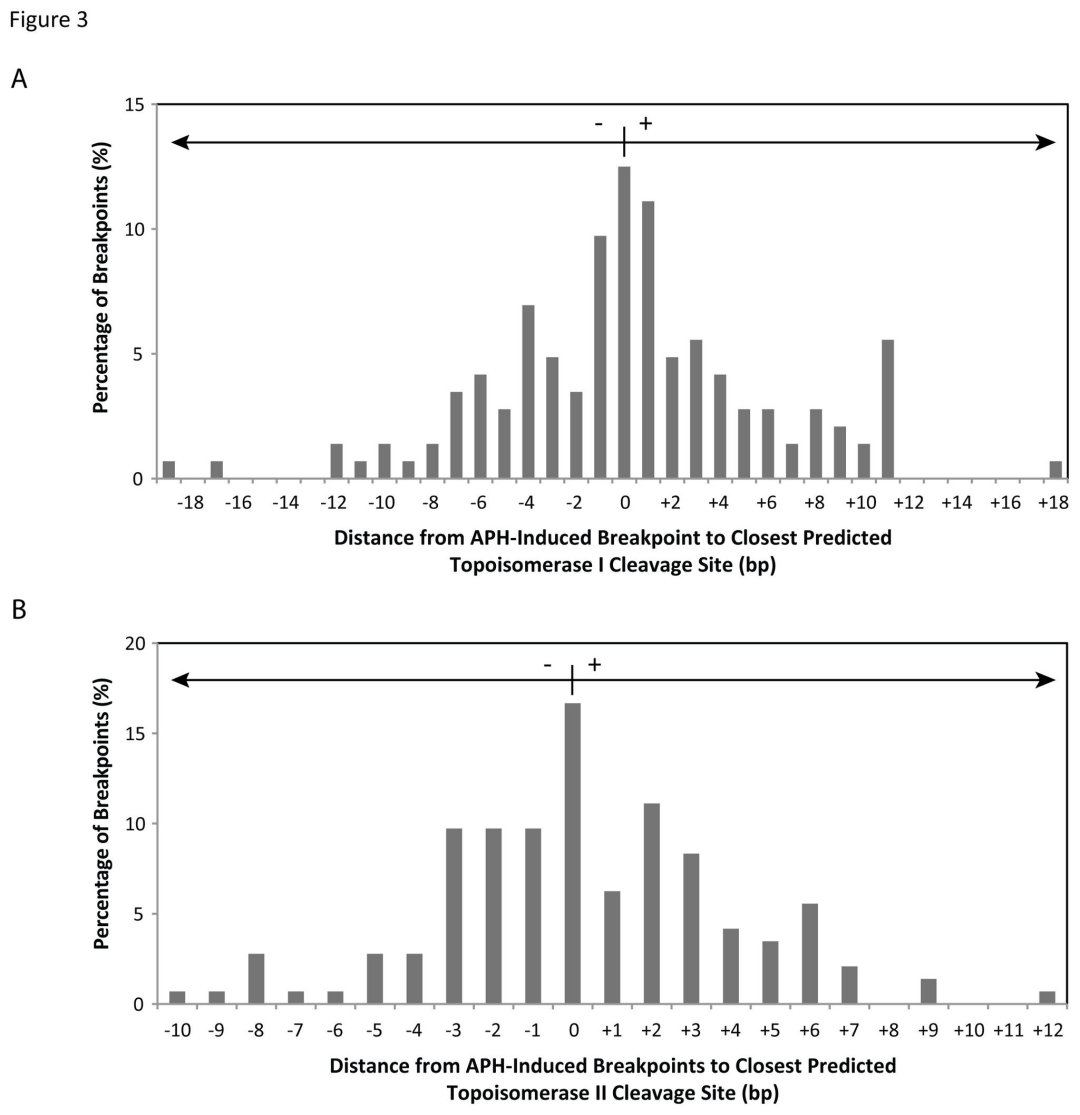
Comparison of 144 APH-induced DNA breakpoints to predicted DNA topoisomerase I and II cleavage sites. (A) Topoisomerase I cleavage sites within *RET* intron 11 were predicted based on the consensus [5’-(A/T/G) (C/G/A) (A/T) (T/C)-3’] immediately upstream of the cleavage site [51], compared to APH-induced DNA breakpoints, and represented as the distance in bp from each APH breakpoint to the closest predicted cleavage site (x axis). A positive distance refers to the closest topoisomase I cleavage site being downstream of the APH breakpoint, and a negative distance being upstream. The percentage of all APH-induced breakpoints is displayed on the y axis. (B) Topoisomerase IIα cleavage sites were predicted using the consensus sequence [5’-(no A) (no T) (A/no C) (-) (C/no A) (-) (-) (-) (-) (no T) (-) (T/no G) (C/ no A) (-)-3’] [52], where breakage occurs between the nucleotides five and six. The locations of APH-induced breakpoints were compared to the predicted sites and represented in the same manner as in (A).

To determine if DNA topoisomerases I and IIα are involved in initiating APH-induced DNA breaks, 144 APH-induced DNA breakpoints were compared to predicted topoisomerase I and IIα cleavage sites. The location of topoisomerase I cleavage sites within *RET* intron 11 were predicted on both DNA strands using the consensus sequence determined by Been et al. [51]. All APH-induced breakpoints were located within 19 bp of a predicted topoisomerase I site, with 76% being within 6 bp (Figure 3A; Table S2). As with topoisomerase I, topoisomerase IIα cleavage sites were predicted within *RET* intron 11 on both strands using the consensus sequence [52] and compared to the APH-induced DNA breaks. All APH-induced breakpoints had a topoisomerase IIα site within 12 bp, with 91% being within 6 bp (Figure 3B; Table S2).

**Figure 4:**
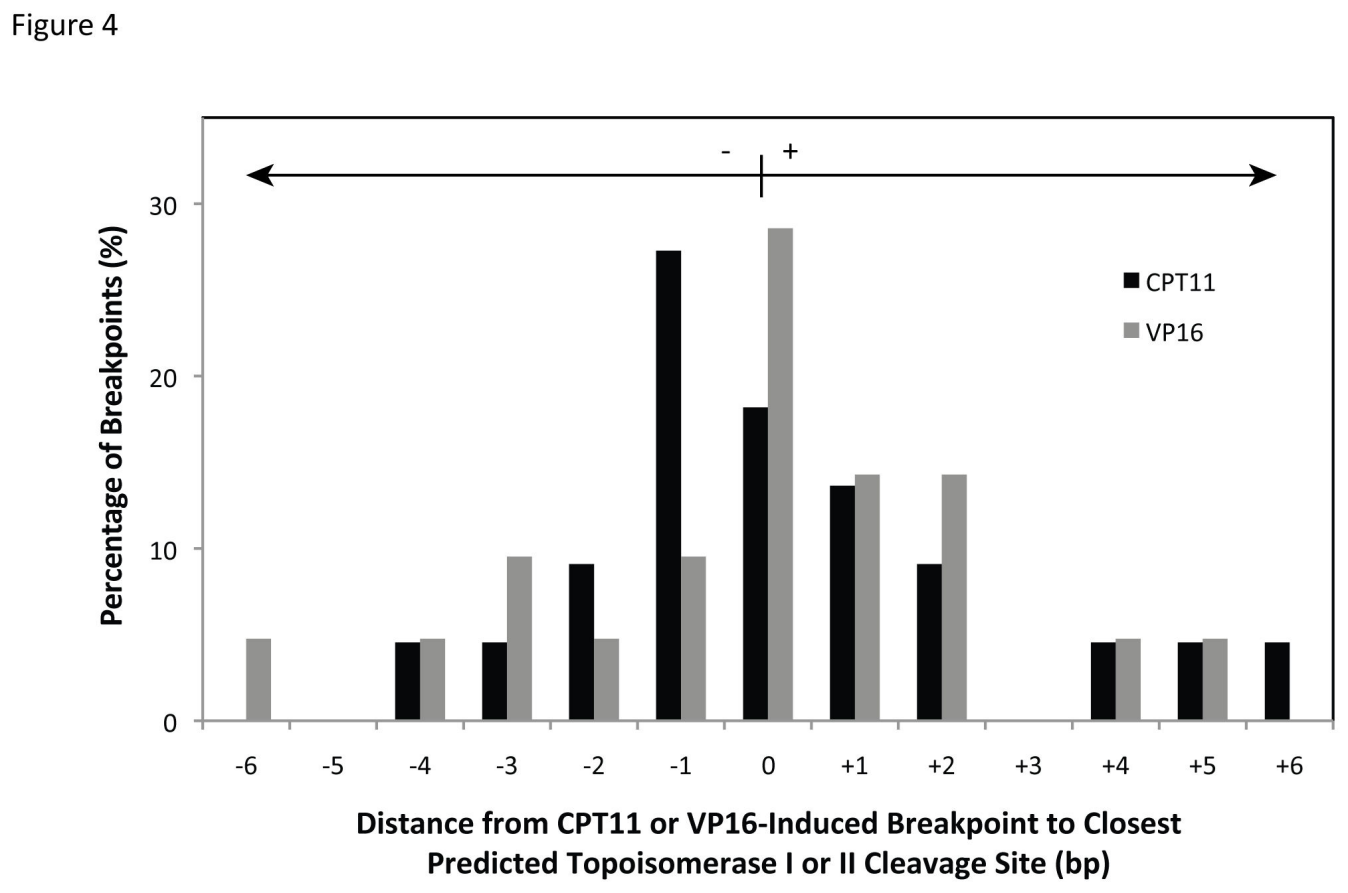
Comparison of CPT 11- and VP-16- induced topoisomerase I and II cleavage to predicted cleavage sites. Topoisomerase I and II DNA cleavage was induced by treatment of HTori-3 cells with 10 µM CPT-11 or VP-16, respectively, for 1.5 hours. The location of CPT 11- (n=22) or VP-16- (n=21) induced DNA cleavage within *RET* intron 11 was detected using LM-PCR and *RET* primer set 1 and compared to either topoisomerase I (CPT 11) or II (VP-16) predicted cleavage sites. A positive distance indicates the predicted cleavage site is downstream of the drug-induced site, and a negative distance indicates the predicted cleavage site is upstream. The percentage of all drug-induced breakpoints is represented on the y axis.

Since the topoisomerase I and IIα consensus sequences are not strictly recognized, we wanted to verify that these enzymes cleave DNA within *RET* intron 11 and that these cleavage sites correspond with the predicted sites. To capture topoisomerase breakage, HTori-3 cells were treated with the topoisomerase poisons CPT 11 and VP-16, which allow topoisomerases I and II, respectively, to cleave DNA but prevent re-ligation [53,54]. HTori-3 cells were exposed to 10 µM CPT-11 or VP-16 for 1.5 hours, treatments known to induce detectable levels of topoisomerase DNA breakage [55,56], after which the DNA was isolated and analyzed for DNA breakage within *RET* intron 11 by LM-PCR using primer set 1. Break frequencies for CPT 11 and VP-16 treated cells were 1.97±1.40 and 1.62 ± 1.20 DNA breaks/100 ng DNA/locus, respectively. A total of 22 breakpoints were sequenced for CPTs 11 and 21 for VP-16 and compared to predicted topoisomerase I or IIα cleavage sites, respectively. Interestingly, 18% of the CPT 11-induced and 29% of the VP-16-induced breakpoints corresponded to a predicted cleavage site (Figure 4). The remaining breakpoints were located within 6 bp of a predicted topoisomerase I or IIα site, suggesting the consensus sequences are not a perfect predictor of topoisomerase breakage.

If we use the criterion set forth by the topoisomerase poisons of being at or within 6 bp of a predicted topoisomerase I or IIα cleavage site, topoisomerase breakage can explain all but one of the APH-induced breakpoints observed. Specifically, 8% are associated with topoisomerase I, 24% with topoisomerase IIα, and 67% with both topoisomerase I and IIα. Together, these results suggest the potential involvement of DNA topoisomerases I and IIα in the initiation of fragile site breakage within *RET* intron 11 following treatment with APH.

#### Comparison of APH-Induced DNA Breakpoints to Sites of Predicted DNA Secondary Structures with Topoisomerase Cleavage Features

Aside from the recognition of consensus sequences in double-stranded DNA, DNA topoisomerases I and II recognize and preferentially cleave single-stranded DNA within regions that form DNA secondary structures. DNA topoisomerase I cleavage of single-stranded DNA requires the formation of a DNA duplex, where cleavage occurs within the duplexed stem of the secondary structure, and the consensus sequence needs only to be approximate [34]. DNA topoisomerase II cleaves DNA hairpins one nucleotide from the 3’-base of the stem, where DNA secondary structure and the presence of a double-stranded/single-stranded DNA junction at the 3’-base of the hairpin, rather than sequence specificity, are the predominant features recognized by the enzyme [35]. Additionally, human topoisomerase IIα recognizes hairpin structures formed within alpha satellite DNA, and cleaves within the single-stranded DNA loop region of the hairpin structure [36]. Recently, using DNA secondary structure-forming analyses, we predicted potential fragile sites on chromosome 10, and among these regions was the *RET* oncogene, including intron 11 [28]. Using an *in vitro* reduplexing assay, we showed that *RET* intron 11 DNA forms significantly greater levels of DNA secondary structure than regions not predicted to possess this ability.

**Figure 5:**
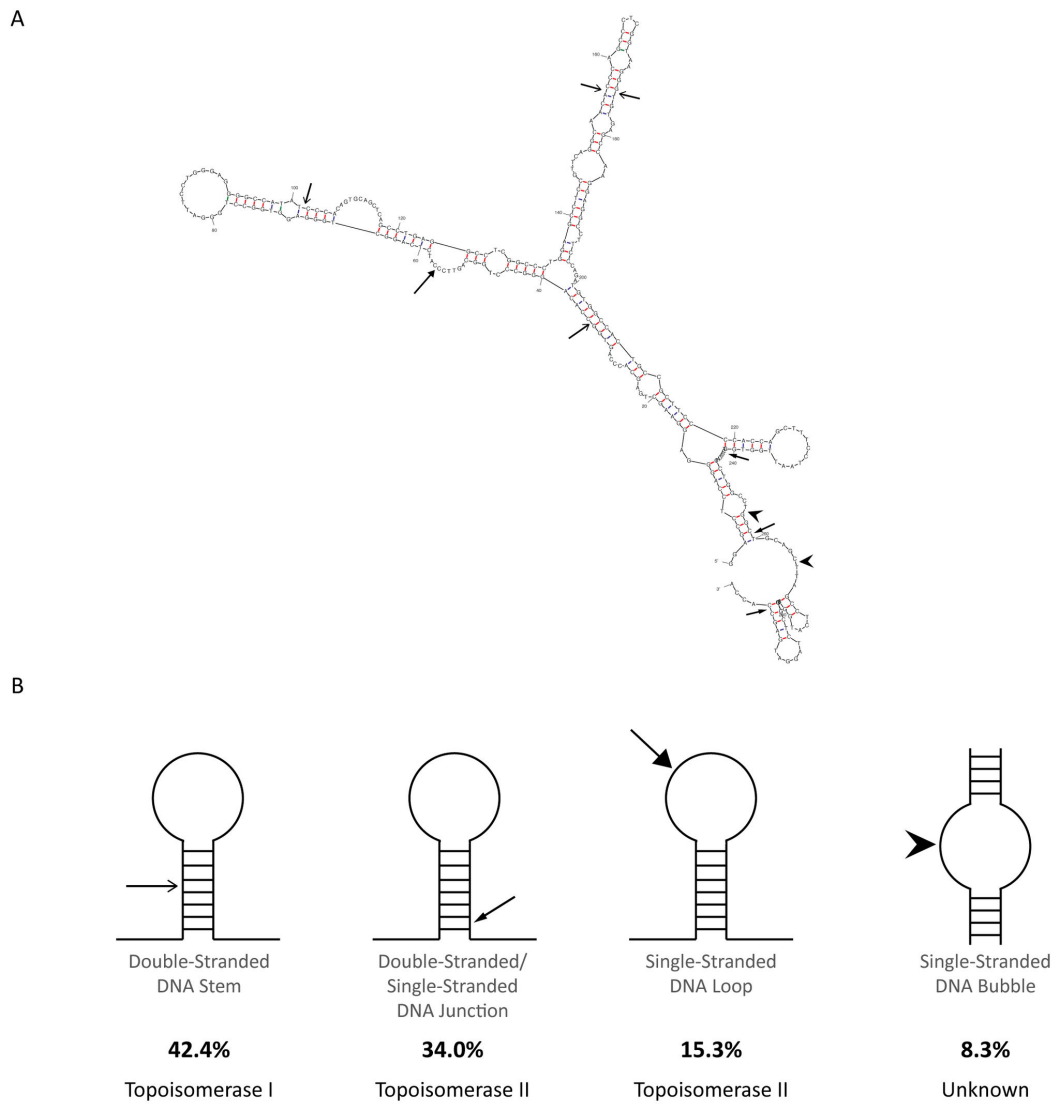
Location of 144 APH-induced RET intron 11 breakpoints on predicted DNA secondary structures. (A) A representative predicted DNA secondary structure is shown, corresponding to the *RET* gene nucleotides 43,610,735 to 43,611,034 (hg37.2), with the locations of APH-induced breakpoints indicated by arrows. The program Mfold was used to predict potential DNA secondary structures within the *RET* intron 11 sequence, by analyzing 300-nt fragments one at a time with a 150-nt shift increment on both DNA strands, and selecting the most energetically favorable structure for each fragment. The location of each APH-induced DNA breakpoint on the predicted secondary structures was analyzed. (B) The percentage of the APH-induced *RET* breakpoints is shown for each DNA secondary structural features recognized by the DNA topoisomerases I and II.

Therefore, to examine correlations between the location of the APH-induced DNA breakpoints and DNA secondary structure formation, potential DNA secondary structures for both DNA strands of *RET* intron 11 were predicted using the program Mfold, analyzing 300-nt segments with 150-nt shift increments and determining the structure with the most favorable free energy value for each DNA segment. Due to the sequence overlap from the segment shift, the location of each DNA breakpoint was analyzed on two potential structures and assigned one structural feature for each breakpoint (Figure 5A; Table S3). Of the 144 APH-induced DNA breakpoints, 61 (42.4%) are located within a predicted double-stranded DNA stem, suggesting the potential involvement of DNA topoisomerase I (Figure 5B). Another 49 breakpoints (34%) are located at a double-stranded/single-stranded DNA junction, and 22 breakpoints (15.3%) are located within a single-stranded DNA loop, suggesting the involvement of DNA topoisomerase II. The remaining 12 breakpoints (8.3%) are located in predicted single-stranded DNA bubbles, which at this time lack a known potential mechanism for DNA cleavage.

These findings provide additional support for the role of DNA topoisomerases I and II in the initiation of APH-induced fragile site breakage within *RET* intron 11. Furthermore, they suggest a mechanistic connection between the formation of DNA secondary structures at fragile sites and the initial DNA breakage events following APH treatment.

#### Topoisomerase Catalytic Inhibitors Decrease APH-Induced DNA Breakage at RET

Since the locations of the APH-induced DNA breakpoints within *RET* intron 11 suggest the potential involvement of topoisomerases I and II in initiating DNA breakage within this region following APH treatment, this hypothesis was directly tested by examining the effect on DNA breakage frequency in HTori-3 cells co-treated with APH and topoisomerase catalytic inhibitors. Topoisomerase catalytic inhibitors block DNA cleavage by the enzyme; therefore, if topoisomerases participate in initiating APH-induced DNA breakage within *RET* intron 11, the catalytic inhibitors would be expected to decrease the rate of APH-induced DNA breakage within this region. Two catalytic inhibitors, betulinic acid and merbarone, were chosen for cell treatments. Betulinic acid (BA) inhibits topoisomerase I DNA cleavage through prevention of topoisomerase I-DNA cleavable complex formation by sequestering topoisomerase I in the nucleoplasm [57]. There have been conflicting reports over the inhibitory effect of BA on topoisomerase IIα [58–60], and no systematic study has been performed to clarify the action of the drug on this enzyme. Merbarone, an inhibitor of DNA topoisomerase II with selectivity for the α over the β isoform [61], inhibits topoisomerase IIα by interacting with the enzyme and preventing DNA scission [62].

**Figure 6:**
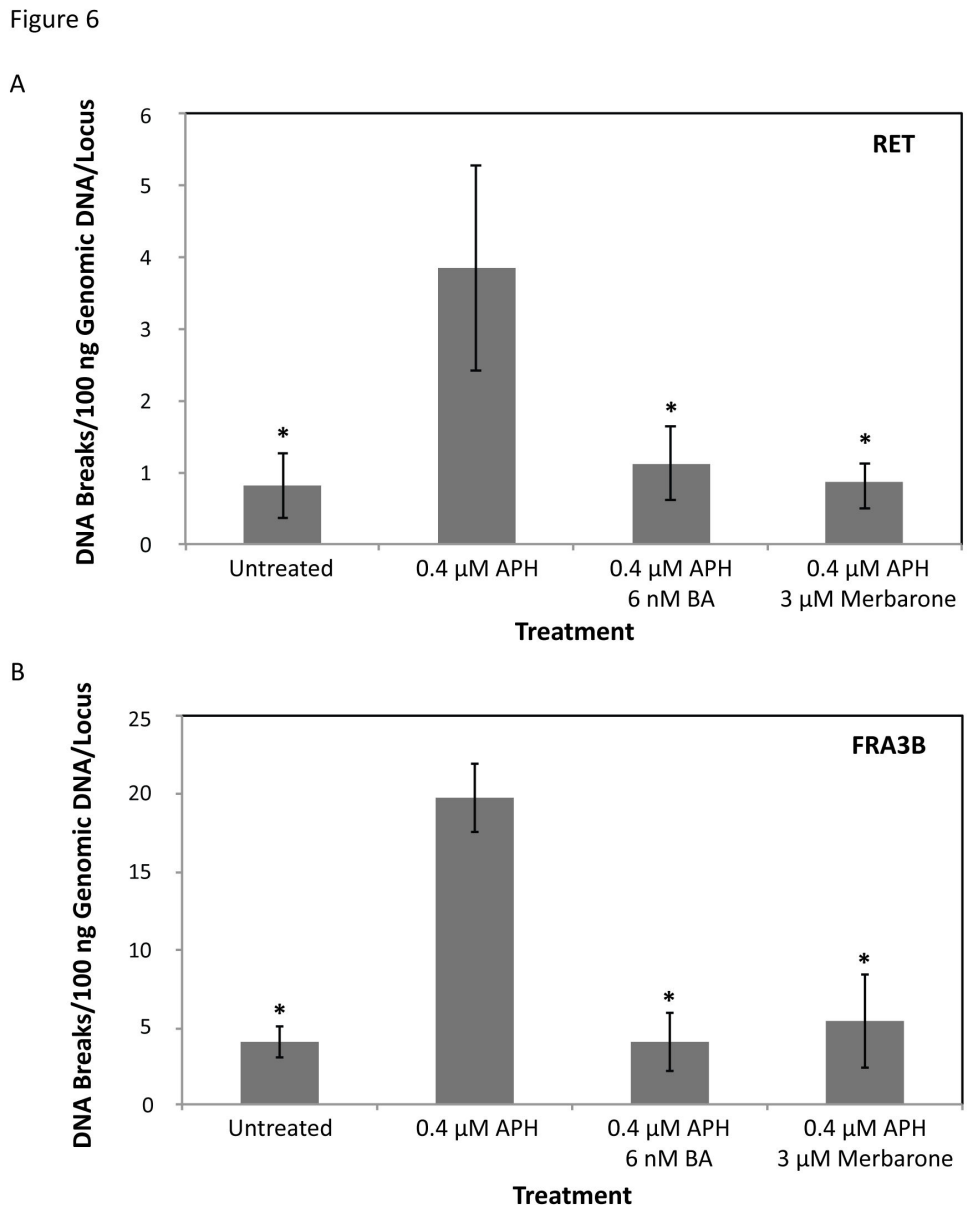
Effects of DNA topoisomerase catalytic inhibitors on the APH-induced common fragile site breakage. (A) HTori-3 cells were treated with 0.4 µM APH in combination with the topoisomerase I and II catalytic inhibitors, 6 nM betulinic acid (BA) or 3 µM merbarone, for 24 hours. LM-PCR was used to detect DNA breaks within *RET* intron 11 using *RET* primer set 1. The frequency of DNA breakage within *RET* intron 11 following 0.4 µM APH treatment combined with 6 nM betulinic acid or 3 µM merbarone significantly decreases compared to APH treatment alone (*P ≤ 3.34E-3), to levels similar to untreated cells. (B) The frequency of DNA breakage within *FHIT* intron 4, located within the APH-induced common fragile site FRA3B, shows a significant increase with 0.4 µM APH treatment. As with *RET* intron 11, the rate of APH-induced DNA breakage within *FHIT* intron 4 significantly decreased when combined with BA or merbarone (*P ≤ 7.21E-6). All data were averaged from 5–7 replicated experiments. All statistical analyses were performed using a two-tailed Student’s T-test. Error bars indicate standard deviations.

Optimal dosages of BA or merbarone were determined in combination with APH treatment in HTori-3 cells, such that significant levels of cell death were not induced and cells were able to replicate their DNA (Figure S2). The cell cycle profile of the cells treated with the combination of chemicals shows a similar pattern as that for aphidicolin treatment alone (Figure S2D). Using these established conditions, HTori-3 cells were treated with 0.4 µM APH and 6 nM BA or 3 µM merbarone for 24 hours. The genomic DNA was then isolated and breakpoint analysis was performed by LM-PCR using *RET* primer set 1 (Figure 6A). Co-treatment of cells with APH and BA or merbarone significantly decreased the level of APH-induced DNA breakage within *RET* intron 11 (1.25 ± 0.58 or 0.97 ± 0.38 breaks/100 ng DNA/locus, *P* = 3.34E-3 or 1.53E-3, respectively, two-tailed Student’s t-test), to levels similar to untreated cells (Figure 6A, Table 1).

Next, the involvement of topoisomerases I and IIα in APH-induced fragile site breakage at the most active common fragile site, FRA3B, was tested by determining the rate of DNA breakage within intron 4 of *FHIT*, a region within FRA3B known to exhibit clustering of APH-induced DNA breakage [41,42]. We previously established that APH treatment in HTori-3 cells results in DNA breakage within this region [9]. In agreement with our previous results, a significant increase in DNA breakage was observed within FRA3B following treatment of HTori-3 cells with 0.4 µM APH for 24 hours (18.40 ± 2.04 breaks/100 ng DNA/locus) compared to untreated (3.75 ± 0.93 breaks/100 ng DNA/locus, *P* = 1.36E-7, two-tailed Student’s t-test; Figure 5C). As was seen in intron 11 of *RET*, when APH treatment was combined with BA or merbarone, the rate of APH-induced DNA breakage significantly decreased (3.75 ± 1.74 or 5.00 ± 2.80 breaks/100 ng DNA/locus, *P* = 5.03E-7 or 7.21E-6, respectively, two-tailed Student’s t-test) to levels similar to untreated cells (Figure 6B). Together, these results confirm that DNA topoisomerases I and IIα are involved in initiating APH-induced DNA breakage within common fragile sites located at *RET* and *FHIT*, and this mechanism may extend to other APH-induced common fragile sites as well. 

## Discussion

In this study, we analyzed the initial events of APH-induced common fragile site breakage within the *RET* oncogene. APH treatment of HTori-3 cells induces significant levels of DNA breakage within intron 11 of *RET*, the breakpoint cluster region found in PTC patients. Previously, we confirmed that APH treatment specifically induces breakage at fragile sites in HTori-3 cells by detecting high levels of breakage within *RET* and *FHIT*, in APH-induced common fragile sites FRA10G and FRA3B, respectively, but not in the non-fragile 12p12.3 region and *G6PD* gene, located within the non-APH inducible rare folate-sensitive fragile sites FRAXF [9]. Using LM-PCR, here we mapped the nucleotide location 144 APH-induced DNA breaks on both strands of *RET* intron 11. All but one of the breakpoints induced by APH were located at or near predicted DNA topoisomerase I and/or IIα cleavage sites (Table S2). Utilizing the DNA secondary structure prediction program Mfold, the locations of these APH-induced breakpoints were compared to predicted DNA secondary structures of the intron 11 sequence. Most breakpoints (91.7%) were located at structural features known to be recognized and preferentially cleaved by topoisomerases I or II (Figure 3). Finally, we confirmed the involvement of topoisomerases I and IIα in APH-induced DNA breakage at the *RET* oncogene by measuring the effect of topoisomerase catalytic inhibitors on the level of APH-induced DNA breakage within intron 11. When catalytic inhibitors BA and merbarone were combined with APH treatment, the frequency of DNA breakage within *RET* intron 11 significantly decreased to levels similar to untreated cells. Furthermore, this effect was also observed at *FHIT* intron 4, confirming the involvement of DNA topoisomerases I and IIα in APH-induced DNA breakage at other common fragile sites as well. Together, these results provide strong evidence that DNA topoisomerases I and IIα have a role in initiating APH-induced DNA breakage at common fragile sites, through recognition and preferential cleavage of DNA secondary structures.

**Figure 7:**
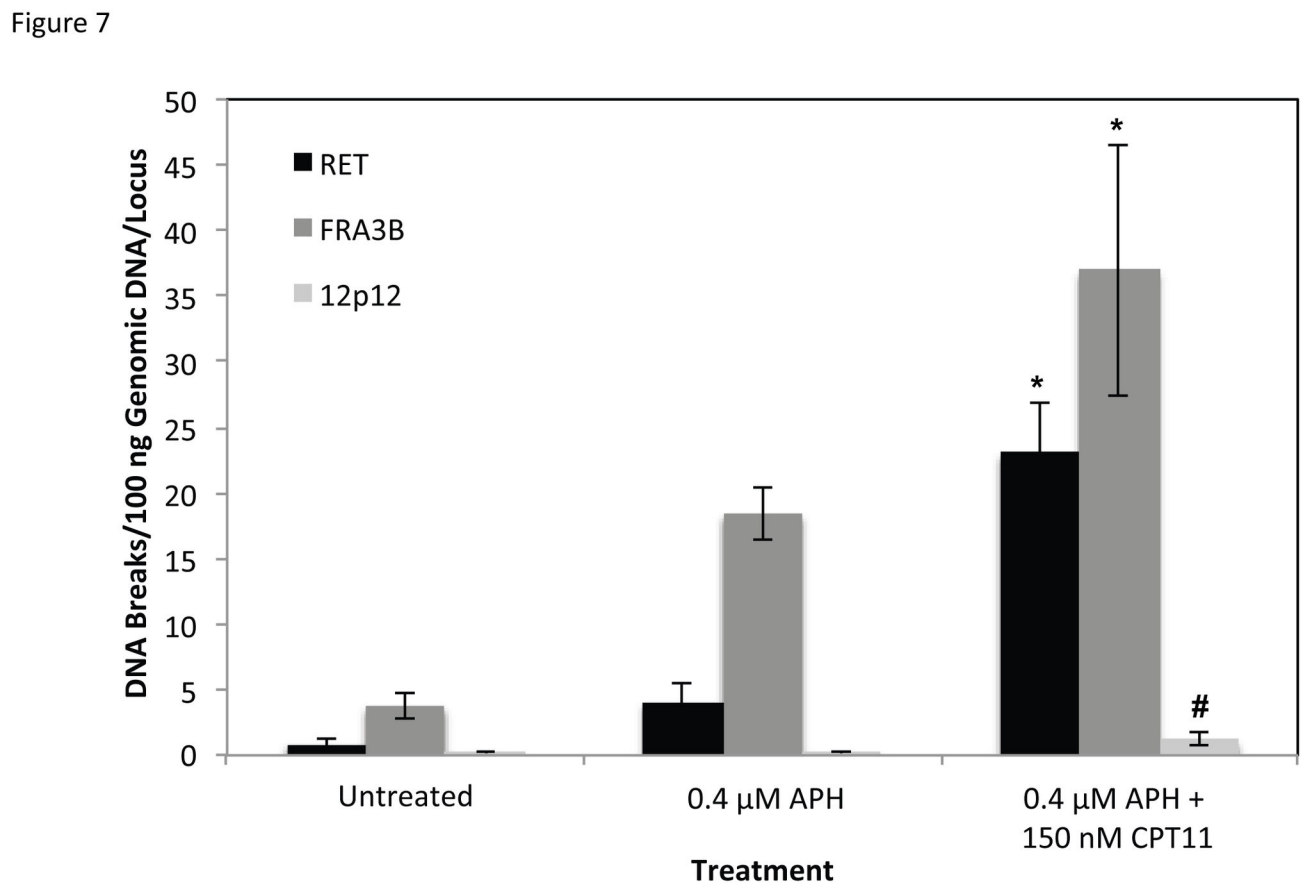
Rate of APH-induced DNA breakage in combination with CPT 11 treatment. Co-treatment of HTori-3 cells with APH and CPT 11 significantly increased the level of DNA breakage within RET intron 11 and FRA3B relative to APH treatment alone (*P ≤ 5.76E-4). This breakage was significantly greater than in the non-fragile 12p12.3 region ( ^#^ P ≤ 2.59E-5). The level of DNA breakage for each treatment was measured using LM-PCR and averaged over at least three independent experiments. Significance was calculated using a two-tailed Student’s T-test. Error bars represent standard deviations.

Previous studies have also implicated DNA topoisomerase I in common fragile site instability. Depletion of topoisomerase I in HCT116 cells significantly increases common fragile site breakage [38]. Arlt et al. observed that co-treatment of cells with APH and the topoisomerase I catalytic inhibitor BA significantly decreased common fragile site breakage, including FRA3B [37]. These results are consistent with our observation that BA significantly decreased APH-induced DNA breakage at *RET* (FRA10G) and *FHIT* (FRA3B) (Figure 6). When Arlt et al. combined APH treatment with the topoisomerase I poison camptothecin (CPT), which prevents topoisomerase I from re-ligating DNA following cleavage, they also observed a significant reduction in common fragile site breakage, including FRA3B [37]. However, when we combined a low dosage (150 nM) of the topoisomerase poison CPT 11 with 0.4 µM APH treatment, we observed a significant increase in APH-induced DNA breakage at *RET* (FRA10G) and *FHIT* (FRA3B) in HTori-3 cells (Figure 7). The differences between our observations and those of Arlt et al. may be attributed to detection methods. We have detected fragile site breakage as single and double strand DNA breaks, while Arlt et al. have detected chromosomal disruptions on metaphase chromosomes. Nevertheless, our data combined with these previous studies provides compelling evidence for DNA topoisomerase involvement in common fragile site breakage.

One model for common fragile site expression is that delayed replication can cause an uncoupling of the helicase complex from the DNA polymerase, resulting in long stretches of single-stranded DNA. Then, at fragile sites this DNA can form stable DNA secondary structures that pause polymerase progression and ultimately result in DNA breakage [10]. The initial events of fragile site breakage remain unclear, but our data here support the involvement of DNA topoisomerases I and IIα in the initiation of APH-induced fragile site breakage. DNA topoisomerases I and IIα participate in replication by maintaining chromosomal structural integrity through transient introduction of DNA breakage. Human topoisomerases I and IIα have been observed at replication origins, and inhibition of topoisomerase I interferes with replication origin firing, indicating these enzymes play a role in replication initiation [63]. Once replication is initiated, DNA is unwound by DNA helicase, resulting in DNA overwinding (positive supercoiling) in front of the replication fork and DNA underwinding (negative supercoiling) behind the replication fork. Positive and negative supercoiling as a result of replication can be removed by both topoisomerases I and IIα [64]. Negative supercoiling behind the replication fork can also result in knots and tangles in the newly replicated DNA, which can be removed by topoisomerase IIα [65]. The presence of these enzymes at the replication fork, their ability to cleave DNA, and their necessity for induction of fragile site breakage at *RET* intron 11 and *FHIT* intron 4 in HTori-3 cells following APH treatment all suggest these enzymes are involved in the initiation of DNA breakage. Furthermore, the location of APH-induced DNA breaks in *RET* intron 11 at DNA secondary structural features recognized by topoisomerases I and II suggests these enzymes may recognize and preferentially cleave these structures while following in front or behind the replication fork scanning for topological changes, or may be recruited separately to these sites.

As with DNA replication, transcription results in positive supercoiling ahead of the transcription bubble and negative supercoiling behind it [66,67]. Furthermore, negative supercoiling enhances the formation of stable RNA-DNA hybrids (R-loops) [68], which are associated with genomic instability and double-strand DNA break formation [69]. Another model for common fragile site breakage is that transcription of long genes at fragile sites results in the formation of stable R-loops due to the collision of transcription and replication machinery, ultimately leading to genomic instability within these regions [29]. DNA topoisomerase I activity is vital during transcription for the removal of positive and negative supercoiling, and thus suppressing R-loop formation [33]. Trinucleotide repeats, including those observed at rare fragile sites, also preferentially form stable R-loops [30–32]. This may be due to the formation of stable DNA secondary structures on the non-template DNA strand [70]. Therefore, as with replication, DNA secondary structure formation at common fragile sites during transcription may result in the formation of stable R-loops and stalled transcription machinery, which may be recognized and preferentially cleaved by DNA topoisomerase I.

RET protein expression in the thyroid is high in neural-crest derived C-cells but not in follicular cells, where *RET/PTC* rearrangements can result in its expression as a fusion protein and lead to PTC. The HTori-3 cell line used in our study is derived from normal human thyroid follicular epithelium [71] and thus does not express RET, which we confirmed by RT-PCR (data not shown). Since active transcription of a gene is required for the transcription-associated model of common fragile site breakage, delayed replication must be responsible for all of the APH-induced fragile site breakage we detected at the *RET* gene. Therefore, the formation of stable R-loops cannot explain breakage at all common fragile sites, further supporting the secondary structure-forming/replication-stalling mechanism of common fragile site breakage.

Cancer is often treated with chemotherapeutic agents that act as DNA topoisomerase poisons. The incidence of secondary primary tumors is on the rise; in the United States, they account for one in six of all newly diagnosed cancers [72], which may be attributed to cancer treatments. Thyroid cancer can occur after treatment of various cancers by chemotherapy, including non-Hodgkin’s lymphoma [73,74] and testicular cancer [75]. Specifically, PTC can occur as a second cancer following treatment of osteosarcoma [76], rhabdomyosarcoma [77], acute lymphoblastic leukemia, neuroblastoma, and Ewing’s sarcoma [78–81] with chemotherapy alone. This suggests that manipulation of normal topoisomerase activity can result in PTC. Since we observe here that perturbed DNA topoisomerase I and II activity can effect fragile site breakage at the *RET* oncogene, more work should be done to investigate the role of topoisomerase poisons and fragile sites in the formation of secondary PTC tumors and other cancers.

These studies show that DNA topoisomerases I and II play a vital role in initiating breakage at APH-induced common fragile sites, providing valuable insight into the initial events of common fragile site breakage. Furthermore, the mechanism by which stable DNA secondary structures form at common fragile sites during delayed replication is supported by our data, and the idea that these structures can be recognized and preferentially cleaved by topoisomerases is presented. Our data provide new mechanistic insights regarding how fragile site breakage at the *RET* oncogene can lead to generation of carcinogenic rearrangements found in thyroid cancer. Since RET rearrangements have also been found in lung cancer, this mechanism may be of broad significance and involved in generation of chromosomal rearrangements in other regions of the genome and in multiple cancer types.

## Supporting Information

Figure S1 **Location of APH-induced breakpoints within intron 11 of *RET* relative to known patient breakpoints.**
(PDF)

Figure S2 **Cell survival of HTori-3 cells following drug treatment.**
(PDF)

Table S1 **Primer and linker sequences for LM-PCR.**
(PDF)

Table S2 **APH‐Induced DNA breakpoints within *RET* Intron 11.**
(PDF)

Table S3 **Location of *RET* Intron 11 APH‐Induced DNA breakpoints on predicted DNA secondary structures.**
(PDF)

## References

[1] Kondo T, Ezzat S, Asa SL (2006) Pathogenetic mechanisms in thyroid follicular-cell neoplasia. Nat Rev Cancer 6: 292-306. doi:10.1038/nrc1836. PubMed: 16557281. 16557281 10.1038/nrc1836

[2] Nikiforov YE, Nikiforova MN (2011) Molecular genetics and diagnosis of thyroid cancer. Nat Rev Endocrinol 7: 569-580. doi:10.1038/nrendo.2011.142. PubMed: 21878896. 21878896 10.1038/nrendo.2011.142

[3] Kohno T, Ichikawa H, Totoki Y, Yasuda K, Hiramoto M et al. (2012) KIF5B-RET fusions in lung adenocarcinoma. Nat Med 18: 375-377. doi:10.1038/nm.2644. PubMed: 22327624. 22327624 10.1038/nm.2644 PMC6430196

[4] Takeuchi K, Soda M, Togashi Y, Suzuki R, Sakata S et al. (2012) RET, ROS1 and ALK fusions in lung cancer. Nat Med 18: 378-381. doi:10.1038/nm.2658. PubMed: 22327623. 22327623 10.1038/nm.2658

[5] Nikiforov YE (2002) RET/PTC rearrangement in thyroid tumors. Endocr Pathol 13: 3-16. doi:10.1385/EP:13:1:03. PubMed: 12114746. 12114746 10.1385/ep:13:1:03

[6] Chen AY, Jemal A, Ward EM (2009) Increasing incidence of differentiated thyroid cancer in the United States, 1988-2005. Cancer 115: 3801-3807. doi:10.1002/cncr.24416. PubMed: 19598221. 19598221 10.1002/cncr.24416

[7] Enewold L, Zhu K, Ron E, Marrogi AJ, Stojadinovic A et al. (2009) Rising thyroid cancer incidence in the United States by demographic and tumor characteristics, 1980-2005. Cancer Epidemiol Biomarkers Prev 18: 784-791. doi:10.1158/1055-9965.EPI-08-0960. PubMed: 19240234. 19240234 10.1158/1055-9965.EPI-08-0960 PMC2676561

[8] Nikiforova MN, Nikiforov YE (2008) Molecular genetics of thyroid cancer: implications for diagnosis, treatment and prognosis. Expert Rev Mol Diagn 8: 83-95. doi:10.1586/14737159.8.1.83. PubMed: 18088233. 18088233 10.1586/14737159.8.1.83

[9] Gandhi M, Dillon LW, Pramanik S, Nikiforov YE, Wang YH (2010) DNA breaks at fragile sites generate oncogenic RET/PTC rearrangements in human thyroid cells. Oncogene 29: 2272-2280. doi:10.1038/onc.2009.502. PubMed: 20101222. 20101222 10.1038/onc.2009.502 PMC2855398

[10] Durkin SG, Glover TW (2007) Chromosome fragile sites. Annu Rev Genet 41: 169-192. doi:10.1146/annurev.genet.41.042007.165900. PubMed: 17608616. 17608616 10.1146/annurev.genet.41.042007.165900

[11] Dillon LW, Burrow AA, Wang YH (2010) DNA instability at chromosomal fragile sites in cancer. Curr Genomics 11: 326-337. doi:10.2174/138920210791616699. PubMed: 21286310. 21286310 10.2174/138920210791616699 PMC2944998

[12] Burrow AA, Williams LE, Pierce LC, Wang YH (2009) Over half of breakpoints in gene pairs involved in cancer-specific recurrent translocations are mapped to human chromosomal fragile sites. BMC Genomics 10: 59. doi:10.1186/1471-2164-10-59. PubMed: 19183484. 19183484 10.1186/1471-2164-10-59 PMC2642838

[13] Bignell GR, Greenman CD, Davies H, Butler AP, Edkins S et al. (2010) Signatures of mutation and selection in the cancer genome. Nature 463: 893-898. doi:10.1038/nature08768. PubMed: 20164919. 20164919 10.1038/nature08768 PMC3145113

[14] Durkin SG, Ragland RL, Arlt MF, Mulle JG, Warren ST et al. (2008) Replication stress induces tumor-like microdeletions in FHIT/FRA3B. Proc Natl Acad Sci U S A 105: 246-251. doi:10.1073/pnas.0708097105. PubMed: 18162546. 18162546 10.1073/pnas.0708097105 PMC2224195

[15] McAvoy S, Ganapathiraju SC, Ducharme-Smith AL, Pritchett JR, Kosari F et al. (2007) Non-random inactivation of large common fragile site genes in different cancers. Cytogenet Genome Res 118: 260-269. doi:10.1159/000108309. PubMed: 18000379. 18000379 10.1159/000108309

[16] Sutherland GR (2003) Rare fragile sites. Cytogenet Genome Res 100: 77-84. doi:10.1159/000072840. PubMed: 14526166. 14526166 10.1159/000072840

[17] Glover TW (2006) Common fragile sites. Cancer Lett 232: 4-12. doi:10.1016/j.canlet.2005.08.032. PubMed: 16229941. 16229941 10.1016/j.canlet.2005.08.032

[18] Cheng CH, Kuchta RD (1993) DNA polymerase epsilon: aphidicolin inhibition and the relationship between polymerase and exonuclease activity. Biochemistry 32: 8568-8574. doi:10.1021/bi00084a025. PubMed: 8395209. 8395209 10.1021/bi00084a025

[19] Glover TW, Berger C, Coyle J, Echo B (1984) DNA polymerase alpha inhibition by aphidicolin induces gaps and breaks at common fragile sites in human chromosomes. Hum Genet 67: 136-142. doi:10.1007/BF00272988. PubMed: 6430783. 6430783 10.1007/BF00272988

[20] Handt O, Baker E, Dayan S, Gartler SM, Woollatt E et al. (2000) Analysis of replication timing at the FRA10B and FRA16B fragile site loci. Chromosome Res 8: 677-688. doi:10.1023/A:1026737203447. PubMed: 11196131. 11196131 10.1023/a:1026737203447

[21] Hellman A, Rahat A, Scherer SW, Darvasi A, Tsui LC et al. (2000) Replication delay along FRA7H, a common fragile site on human chromosome 7, leads to chromosomal instability. Mol Cell Biol 20: 4420-4427. doi:10.1128/MCB.20.12.4420-4427.2000. PubMed: 10825205. 10825205 10.1128/mcb.20.12.4420-4427.2000 PMC85809

[22] Le Beau, Rassool FV, Neilly ME, Espinosa R 3rd, Glover TW et al. (1998) Replication of a common fragile site, FRA3B, occurs late in S phase and is delayed further upon induction: implications for the mechanism of fragile site induction. Hum Mol Genet 7: 755-761. doi:10.1093/hmg/7.4.755. PubMed: 9499431. 9499431 10.1093/hmg/7.4.755

[23] Pelliccia F, Bosco N, Curatolo A, Rocchi A (2008) Replication timing of two human common fragile sites: FRA1H and FRA2G. Cytogenet Genome Res 121: 196-200. doi:10.1159/000138885. PubMed: 18758159. 18758159 10.1159/000138885

[24] Mishmar D, Rahat A, Scherer SW, Nyakatura G, Hinzmann B et al. (1998) Molecular characterization of a common fragile site (FRA7H) on human chromosome 7 by the cloning of a simian virus 40 integration site. Proc Natl Acad Sci U S A 95: 8141-8146. doi:10.1073/pnas.95.14.8141. PubMed: 9653154. 9653154 10.1073/pnas.95.14.8141 PMC20943

[25] Zlotorynski E, Rahat A, Skaug J, Ben-Porat N, Ozeri E et al. (2003) Molecular basis for expression of common and rare fragile sites. Mol Cell Biol 23: 7143-7151. doi:10.1128/MCB.23.20.7143-7151.2003. PubMed: 14517285. 14517285 10.1128/MCB.23.20.7143-7151.2003 PMC230307

[26] Zhang H, Freudenreich CH (2007) An AT-rich sequence in human common fragile site FRA16D causes fork stalling and chromosome breakage in S. cervisiae. Mol Cell 27: 367-379. doi:10.1016/j.molcel.2007.06.012. PubMed: 17679088. 17679088 10.1016/j.molcel.2007.06.012 PMC2144737

[27] Shah SN, Opresko PL, Meng X, Lee MY, Eckert KA (2010) DNA structure and the Werner protein modulate human DNA polymerase delta-dependent replication dynamics within the common fragile site FRA16D. Nucleic Acids Res 38: 1149-1162. doi:10.1093/nar/gkp1131. PubMed: 19969545. 19969545 10.1093/nar/gkp1131 PMC2831333

[28] Dillon LW, Pierce LC, Ng MC, Wang YH (2013) Role of DNA secondary structures in fragile site breakage along human chromosome 10. Hum Mol Genet, 1 12/ [Epub ahead of print] PubMed: 23297364. 10.1093/hmg/dds561 PMC3596854 23297364

[29] Helmrich A, Ballarino M, Tora L (2011) Collisions between replication and transcription complexes cause common fragile site instability at the longest human genes. Mol Cell 44: 966-977. doi:10.1016/j.molcel.2011.10.013. PubMed: 22195969. 22195969 10.1016/j.molcel.2011.10.013

[30] Grabczyk E, Mancuso M, Sammarco MC (2007) A persistent RNA.DNA hybrid formed by transcription of the Friedreich ataxia triplet repeat in live bacteria, and by T7 RNAP in vitro. Nucleic Acids Res 35: 5351-5359. doi:10.1093/nar/gkm589. PubMed: 17693431. 17693431 10.1093/nar/gkm589 PMC2018641

[31] Lin Y, Dent SY, Wilson JH, Wells RD, Napierala M (2010) R loops stimulate genetic instability of CTG.CAG repeats. Proc Natl Acad Sci U S A 107: 692-697. doi:10.1073/pnas.0909740107. PubMed: 20080737. 20080737 10.1073/pnas.0909740107 PMC2818888

[32] Reddy K, Tam M, Bowater RP, Barber M, Tomlinson M et al. (2011) Determinants of R-loop formation at convergent bidirectionally transcribed trinucleotide repeats. Nucleic Acids Res 39: 1749-1762. doi:10.1093/nar/gkq935. PubMed: 21051337. 21051337 10.1093/nar/gkq935 PMC3061079

[33] Vos SM, Tretter EM, Schmidt BH, Berger JM (2011) All tangled up: how cells direct, manage and exploit topoisomerase function. Nat Rev Mol Cell Biol 12: 827-841. doi:10.1038/nrm3228. PubMed: 22108601. 22108601 10.1038/nrm3228 PMC4351964

[34] Been MD, Champoux JJ (1984) Breakage of single-stranded DNA by eukaryotic type 1 topoisomerase occurs only at regions with the potential for base-pairing. J Mol Biol 180: 515-531. doi:10.1016/0022-2836(84)90025-1. PubMed: 6098684. 6098684 10.1016/0022-2836(84)90025-1

[35] Froelich-Ammon SJ, Gale KC, Osheroff N (1994) Site-specific cleavage of a DNA hairpin by topoisomerase II. DNA secondary structure as a determinant of enzyme recognition/cleavage. J Biol Chem 269: 7719-7725. PubMed: 8125998. 8125998

[36] Jonstrup AT, Thomsen T, Wang Y, Knudsen BR, Koch J et al. (2008) Hairpin structures formed by alpha satellite DNA of human centromeres are cleaved by human topoisomerase IIalpha. Nucleic Acids Res 36: 6165-6174. doi:10.1093/nar/gkn640. PubMed: 18824478. 18824478 10.1093/nar/gkn640 PMC2577340

[37] Arlt MF, Glover TW (2010) Inhibition of topoisomerase I prevents chromosome breakage at common fragile sites. DNA Repair (Amst) 9: 678-689. doi:10.1016/j.dnarep.2010.03.005. PubMed: 20413351. 20413351 10.1016/j.dnarep.2010.03.005 PMC2896008

[38] Tuduri S, Crabbé L, Conti C, Tourrière H, Holtgreve-Grez H et al. (2009) Topoisomerase I suppresses genomic instability by preventing interference between replication and transcription. Nat Cell Biol 11: 1315-1324. doi:10.1038/ncb1984. PubMed: 19838172. 19838172 10.1038/ncb1984 PMC2912930

[39] Smanik PA, Furminger TL, Mazzaferri EL, Jhiang SM (1995) Breakpoint characterization of the ret/PTC oncogene in human papillary thyroid carcinoma. Hum Mol Genet 4: 2313-2318. doi:10.1093/hmg/4.12.2313. PubMed: 8634704. 8634704 10.1093/hmg/4.12.2313

[40] Caudill CM, Zhu Z, Ciampi R, Stringer JR, Nikiforov YE (2005) Dose-dependent generation of RET/PTC in human thyroid cells after in vitro exposure to gamma-radiation: a model of carcinogenic chromosomal rearrangement induced by ionizing radiation. J Clin Endocrinol Metab 90: 2364-2369. doi:10.1210/jc.2004-1811. PubMed: 15671095. 15671095 10.1210/jc.2004-1811

[41] Mulvihill DJ, Wang YH (2004) Two breakpoint clusters at fragile site FRA3B form phased nucleosomes. Genome Res 14: 1350-1357. doi:10.1101/gr.2304404. PubMed: 15231750. 15231750 10.1101/gr.2304404 PMC442151

[42] Corbin S, Neilly ME, Espinosa R 3rd, Davis EM, McKeithan TW et al. (2002) Identification of unstable sequences within the common fragile site at 3p14.2: implications for the mechanism of deletions within fragile histidine triad gene/common fragile site at 3p14.2 in tumors. Cancer Res 62: 3477-3484. PubMed: 12067991. 12067991

[43] Sambrook J, Fritsch EF, Maniatis T (1989) Molecular cloning : a laboratory manual. Cold Spring Harbor, NY: Cold Spring Harbor Laboratory.

[44] Zuker M (2003) Mfold web server for nucleic acid folding and hybridization prediction. Nucleic Acids Res 31: 3406-3415. doi:10.1093/nar/gkg595. PubMed: 12824337. 12824337 10.1093/nar/gkg595 PMC169194

[45] Anderson S, DePamphilis ML (1979) Metabolism of Okazaki fragments during simian virus 40 DNA replication. J Biol Chem 254: 11495-11504. PubMed: 227871. 227871

[46] Hay RT, DePamphilis ML (1982) Initiation of SV40 DNA replication in vivo: location and structure of 5' ends of DNA synthesized in the ori region. Cell 28: 767-779. doi:10.1016/0092-8674(82)90056-3. PubMed: 6178514. 6178514 10.1016/0092-8674(82)90056-3

[47] Bongarzone I, Butti MG, Fugazzola L, Pacini F, Pinchera A et al. (1997) Comparison of the breakpoint regions of ELE1 and RET genes involved in the generation of RET/PTC3 oncogene in sporadic and in radiation-associated papillary thyroid carcinomas. Genomics 42: 252-259. doi:10.1006/geno.1997.4685. PubMed: 9192845. 9192845 10.1006/geno.1997.4685

[48] Jhiang SM, Caruso DR, Gilmore E, Ishizaka Y, Tahira T et al. (1992) Detection of the PTC/retTPC oncogene in human thyroid cancers. Oncogene 7: 1331-1337. PubMed: 1620547. 1620547

[49] Klugbauer S, Pfeiffer P, Gassenhuber H, Beimfohr C, Rabes HM (2001) RET rearrangements in radiation-induced papillary thyroid carcinomas: high prevalence of topoisomerase I sites at breakpoints and microhomology-mediated end joining in ELE1 and RET chimeric genes. Genomics 73: 149-160. doi:10.1006/geno.2000.6434. PubMed: 11318605. 11318605 10.1006/geno.2000.6434

[50] Nikiforov YE, Koshoffer A, Nikiforova M, Stringer J, Fagin JA (1999) Chromosomal breakpoint positions suggest a direct role for radiation in inducing illegitimate recombination between the ELE1 and RET genes in radiation-induced thyroid carcinomas. Oncogene 18: 6330-6334. doi:10.1038/sj.onc.1203019. PubMed: 10597232. 10597232 10.1038/sj.onc.1203019

[51] Been MD, Burgess RR, Champoux JJ (1984) Nucleotide sequence preference at rat liver and wheat germ type 1 DNA topoisomerase breakage sites in duplex SV40 DNA. Nucleic Acids Res 12: 3097-3114. doi:10.1093/nar/12.7.3097. PubMed: 6326051. 6326051 10.1093/nar/12.7.3097 PMC318732

[52] Capranico G, Binaschi M (1998) DNA sequence selectivity of topoisomerases and topoisomerase poisons. Biochim Biophys Acta 1400: 185-194. doi:10.1016/S0167-4781(98)00135-3. PubMed: 9748568. 9748568 10.1016/s0167-4781(98)00135-3

[53] Garcia-Carbonero R, Supko JG (2002) Current perspectives on the clinical experience, pharmacology, and continued development of the camptothecins. Clin Cancer Res 8: 641-661. PubMed: 11895891. 11895891

[54] Hande KR (2008) Topoisomerase II inhibitors. Update Cancer Therapeutics 3: 13-26. doi:10.1016/j.uct.2008.02.001. PubMed: 16110609153387421270320311686011.

[55] Long BH, Musial ST, Brattain MG (1985) Single- and double-strand DNA breakage and repair in human lung adenocarcinoma cells exposed to etoposide and teniposide. Cancer Res 45: 3106-3112. PubMed: 3839166. 3839166

[56] Pondarré C, Strumberg D, Fujimori A, Torres-León R, Pommier Y (1997) In vivo sequencing of camptothecin-induced topoisomerase I cleavage sites in human colon carcinoma cells. Nucleic Acids Res 25: 4111-4116. doi:10.1093/nar/25.20.4111. PubMed: 9321666. 9321666 10.1093/nar/25.20.4111 PMC147024

[57] Ganguly A, Das B, Roy A, Sen N, Dasgupta SB et al. (2007) Betulinic acid, a catalytic inhibitor of topoisomerase I, inhibits reactive oxygen species-mediated apoptotic topoisomerase I-DNA cleavable complex formation in prostate cancer cells but does not affect the process of cell death. Cancer Res 67: 11848-11858. doi:10.1158/0008-5472.CAN-07-1615. PubMed: 18089815. 18089815 10.1158/0008-5472.CAN-07-1615

[58] Bar FM, Khanfar MA, Elnagar AY, Liu H, Zaghloul AM et al. (2009) Rational design and semisynthesis of betulinic acid analogues as potent topoisomerase inhibitors. J Nat Prod 72: 1643-1650. doi:10.1021/np900312u. PubMed: 19691293. 19691293 10.1021/np900312u

[59] Syrovets T, Büchele B, Gedig E, Slupsky JR, Simmet T (2000) Acetyl-boswellic acids are novel catalytic inhibitors of human topoisomerases I and IIalpha. Mol Pharmacol 58: 71-81. PubMed: 10860928. 10860928 10.1124/mol.58.1.71

[60] Wada S, Tanaka R (2005) Betulinic acid and its derivatives, potent DNA topoisomerase II inhibitors, from the bark of Bischofia javanica. Chem Biodivers 2: 689-694. doi:10.1002/cbdv.200590045. PubMed: 17192012. 17192012 10.1002/cbdv.200590045

[61] Drake FH, Hofmann GA, Mong SM, Bartus JO, Hertzberg RP et al. (1989) In vitro and intracellular inhibition of topoisomerase II by the antitumor agent merbarone. Cancer Res 49: 2578-2583. PubMed: 2540903. 2540903

[62] Fortune JM, Osheroff N (1998) Merbarone inhibits the catalytic activity of human topoisomerase IIalpha by blocking DNA cleavage. J Biol Chem 273: 17643-17650. doi:10.1074/jbc.273.28.17643. PubMed: 9651360. 9651360 10.1074/jbc.273.28.17643

[63] Abdurashidova G, Radulescu S, Sandoval O, Zahariev S, Danailov MB et al. (2007) Functional interactions of DNA topoisomerases with a human replication origin. EMBO J 26: 998-1009. doi:10.1038/sj.emboj.7601578. PubMed: 17290216. 17290216 10.1038/sj.emboj.7601578 PMC1852844

[64] Wang JC (2002) Cellular roles of DNA topoisomerases: a molecular perspective. Nat Rev Mol Cell Biol 3: 430-440. doi:10.1038/nrm831. PubMed: 12042765. 12042765 10.1038/nrm831

[65] Postow L, Crisona NJ, Peter BJ, Hardy CD, Cozzarelli NR (2001) Topological challenges to DNA replication: conformations at the fork. Proc Natl Acad Sci U S A 98: 8219-8226. doi:10.1073/pnas.111006998. PubMed: 11459956. 11459956 10.1073/pnas.111006998 PMC37424

[66] Liu LF, Wang JC (1987) Supercoiling of the DNA template during transcription. Proc Natl Acad Sci U S A 84: 7024-7027. doi:10.1073/pnas.84.20.7024. PubMed: 2823250. 2823250 10.1073/pnas.84.20.7024 PMC299221

[67] Wu HY, Shyy SH, Wang JC, Liu LF (1988) Transcription generates positively and negatively supercoiled domains in the template. Cell 53: 433-440. doi:10.1016/0092-8674(88)90163-8. PubMed: 2835168. 2835168 10.1016/0092-8674(88)90163-8

[68] Drolet M, Bi X, Liu LF (1994) Hypernegative supercoiling of the DNA template during transcription elongation in vitro. J Biol Chem 269: 2068-2074. PubMed: 8294458. 8294458

[69] Li X, Manley JL (2005) Inactivation of the SR protein splicing factor ASF/SF2 results in genomic instability. Cell 122: 365-378. doi:10.1016/j.cell.2005.06.008. PubMed: 16096057. 16096057 10.1016/j.cell.2005.06.008

[70] Lin Y, Wilson JH (2011) Transcription-induced DNA toxicity at trinucleotide repeats: double bubble is trouble. Cell Cycle 10: 611-618. doi:10.4161/cc.10.4.14729. PubMed: 21293182. 21293182 10.4161/cc.10.4.14729 PMC3173998

[71] Lemoine NR, Mayall ES, Jones T, Sheer D, McDermid S et al. (1989) Characterisation of human thyroid epithelial cells immortalised in vitro by simian virus 40 DNA transfection. Br J Cancer 60: 897-903. doi:10.1038/bjc.1989.387. PubMed: 2557880. 2557880 10.1038/bjc.1989.387 PMC2247263

[72] Allan JM, Travis LB (2005) Mechanisms of therapy-related carcinogenesis. Nat Rev Cancer 5: 943-955. doi:10.1038/nrc1749. PubMed: 16294218. 16294218 10.1038/nrc1749

[73] Boffetta P, Kaldor JM (1994) Secondary malignancies following cancer chemotherapy. Acta Oncol 33: 591-598. doi:10.3109/02841869409121767. PubMed: 7946433. 7946433 10.3109/02841869409121767

[74] Swerdlow AJ, Douglas AJ, Vaughan Hudson G, Vaughan Hudson B, MacLennan KA (1993) Risk of second primary cancer after Hodgkin’s disease in patients in the British National Lymphoma Investigation: relationships to host factors, histology and stage of Hodgkin’s disease, and splenectomy. Br J Cancer 68: 1006-1011. doi:10.1038/bjc.1993.470. PubMed: 8217588. 8217588 10.1038/bjc.1993.470 PMC1968752

[75] Travis LB, Curtis RE, Storm H, Hall P, Holowaty E et al. (1997) Risk of second malignant neoplasms among long-term survivors of testicular cancer. J Natl Cancer Inst 89: 1429-1439. doi:10.1093/jnci/89.19.1429. PubMed: 9326912. 9326912 10.1093/jnci/89.19.1429

[76] Kim MS, Sim YS, Lee SY, Jeon DG (2008) Secondary thyroid papillary carcinoma in osteosarcoma patients: report of two cases. J Korean Med Sci 23: 149-152. doi:10.3346/jkms.2008.23.1.149. PubMed: 18303218. 18303218 10.3346/jkms.2008.23.1.149 PMC2526477

[77] Venkitaraman R, Affolter A, Ahmed M, Thomas V, Pritchard-Jones K et al. (2008) Childhood papillary thyroid cancer as second malignancy after successful treatment of rhabdomyosarcoma. Acta Oncol 47: 469-472. doi:10.1080/02841860701864676. PubMed: 18348005. 18348005 10.1080/02841860701864676

[78] de Vathaire F, Hawkins M, Campbell S, Oberlin O, Raquin MA et al. (1999) Second malignant neoplasms after a first cancer in childhood: temporal pattern of risk according to type of treatment. Br J Cancer 79: 1884-1893. doi:10.1038/sj.bjc.6690300. PubMed: 10206309. 10206309 10.1038/sj.bjc.6690300 PMC2362818

[79] Gow KW, Lensing S, Hill DA, Krasin MJ, McCarville MB et al. (2003) Thyroid carcinoma presenting in childhood or after treatment of childhood malignancies: An institutional experience and review of the literature. J Pediatr Surg 38: 1574-1580. doi:10.1016/S0022-3468(03)00563-3. PubMed: 14614703. 14614703 10.1016/s0022-3468(03)00563-3

[80] Smith MB, Xue H, Strong L, Takahashi H, Jaffe N et al. (1993) Forty-year experience with second malignancies after treatment of childhood cancer: analysis of outcome following the development of the second malignancy. J Pediatr Surg 28: 1342-1348; discussion 1348-1349 doi:10.1016/S0022-3468(05)80325-2. PubMed: 8263699. 8263699 10.1016/s0022-3468(05)80325-2

[81] Vane D, King DR, Boles ET Jr. (1984) Secondary thyroid neoplasms in pediatric cancer patients: increased risk with improved survival. J Pediatr Surg 19: 855-860. doi:10.1016/S0022-3468(84)80384-X. PubMed: 6097662. 6097662 10.1016/s0022-3468(84)80384-x

