# Supplementary material for: DNA Topoisomerases Participate in Fragility of the Oncogene RET
Source: PLoS One. 2013 Sep 11;8(9):e75741. doi: 10.1371/journal.pone.0075741 (PMC3770543; doi:10.1371/journal.pone.0075741)
Supplement: Figure S1 — Location of APH-induced breakpoints within intron 11 of RET relative to known patient breakpoints. (PDF) [file pone.0075741.s001.pdf]

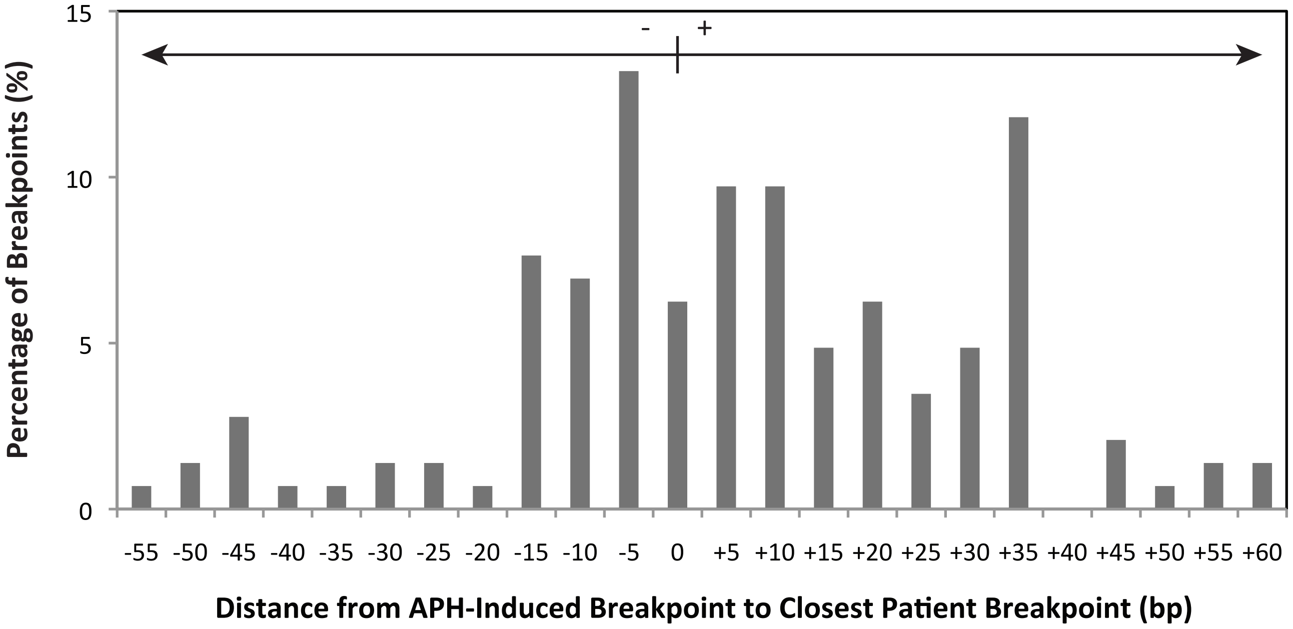

Figure S1. Location of APH-induced breakpoints within intron 11 of *RET* relative to known patient breakpoints. The nucleotide location of previously reported fusion points observed in PTC patients with *RET/PTC1* or *RET/PTC3* translocations [39, 47-50] were compared to 144 APH-induced breakpoints. The distance range is represented on the x axis, where a negative position refers to the closest patient breakpoint being upstream of the APH-induced breakpoint and a positive being downstream, and the percentage of the total APH-induced breakpoints contained within the distance range is displayed on the y axis.
