# Supplementary material for: DNA Topoisomerases Participate in Fragility of the Oncogene RET
Source: PLoS One. 2013 Sep 11;8(9):e75741. doi: 10.1371/journal.pone.0075741 (PMC3770543; doi:10.1371/journal.pone.0075741)
Supplement: Figure S2 — Cell survival of HTori-3 cells following drug treatment. (PDF) [file pone.0075741.s002.pdf]

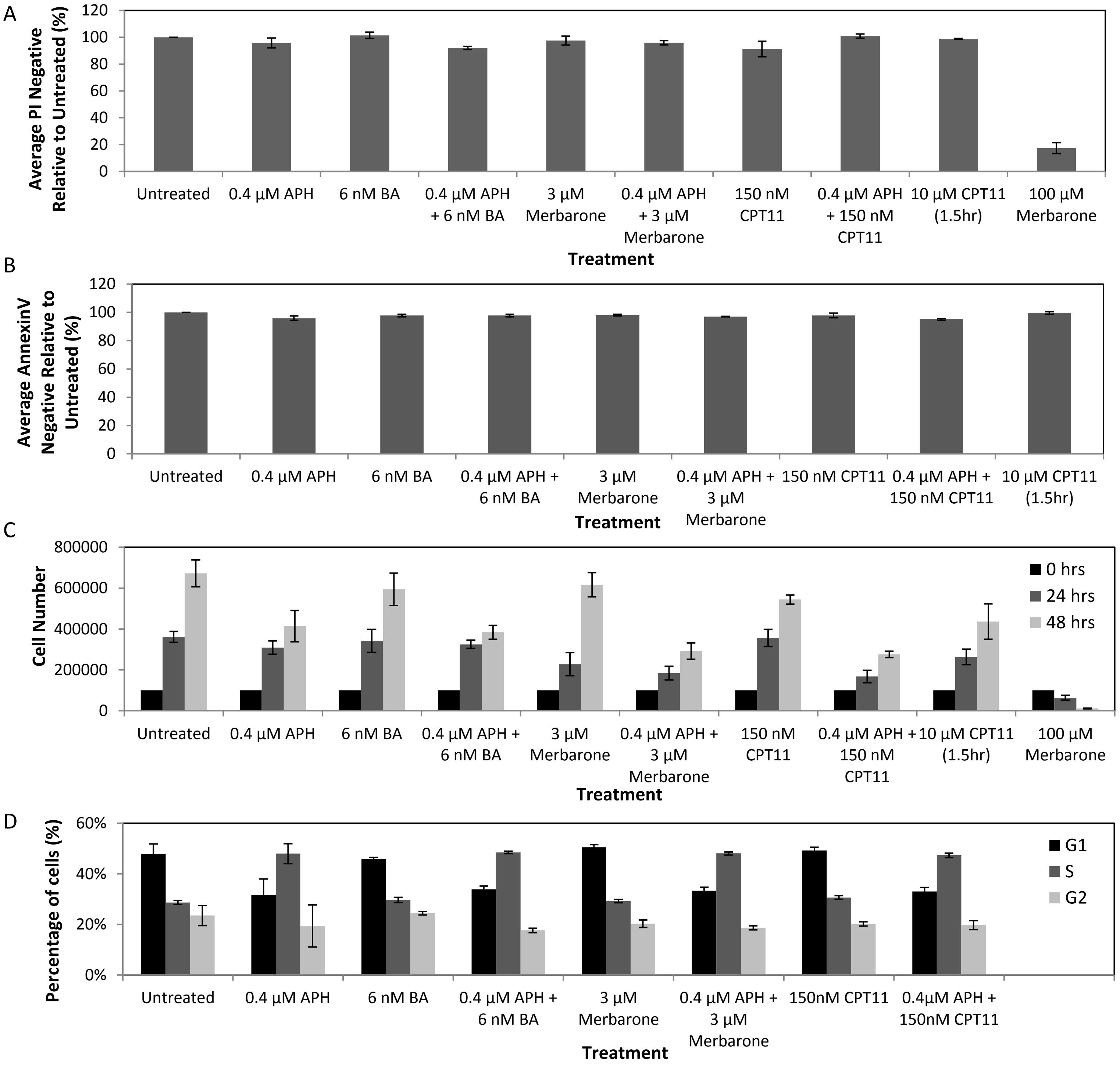

Figure S2. Cell survival of HTori-3 cells following drug treatment. (A) The level of HTori-3 cell death following treatment with APH and/or topoisomerase inhibitor drugs was determined using a propidium iodide (PI) stain and measured using flow cytometry. The percentage of live cells (PI negative) relative to untreated cells was determined for each treatment. Treatment with 100µM Merbarone induced significant cell death. (B) The number of HTori-3 cells actively undergoing apoptosis following treatment with APH and/or topoisomerase inhibitor drugs was determined using AnnexinV stain and measured using flow cytometry. The percentage of non-apoptotic cells (AnnexinV negative) relative to untreated cells was determined. (C) The level of cell growth following treatment with APH and/or topoisomerase inhibitor drugs was determined by quantification using a hemocytometer. Cells were counted at the time of plating (0 hrs), after 24 hour treatment (24 hrs), or allowed to recover for an additional 24 hours after chemicals had been removed (48 hrs). (D) Cell cycle profile was determined for each treatment using PI stain and measured using flow cytometry. All assays represent the average of at least four experimental replicates with error bars representing standard deviations.
