# Supplementary material for: DNA Topoisomerases Participate in Fragility of the Oncogene RET
Source: PLoS One. 2013 Sep 11;8(9):e75741. doi: 10.1371/journal.pone.0075741 (PMC3770543; doi:10.1371/journal.pone.0075741)
Supplement: Table S1 — Primer and linker sequences for LM-PCR. (PDF) [file pone.0075741.s003.pdf]

Table S1. Primer and Linker Sequences for LM-PCR.

Region	Primer Set	Primer Name	Primer Sequence[a]
**RET**	1	RET-7	5’- BBCAGCATCTTCACGGCCACCGTGG-3’
		RET-R1b	5’- TATCCTGCTCTGCCTTTCAGATGG-3’
		RET-R1	5’-AGTTCTTCCGAGATTCC-3’
	2	RET-8	5'-BBCACATGAGGGCGACATCAACCTG-3'
		RET-9	5'-ACCTGTTTACCACACTCTAGAGAC-3'
		RET-10	5'-CCTCTCAAATACTGAGGTTGAGTC-3'
	3	RET-14	5'-BBTACCACAAGTTTGCCCACAAGCCACCC-3'
		RET-15	5'-CCCGGTCAGCTACTCCTCTTCC-3'
		RET-16	5'-TCTCCGTGGATGCCTTCAAGAT-3'
	4	RET-17	5'-BBGCTCTAGGATGAGCCACCAGAGTCC-3'
		RET-18	5'-CCAGGAAGGCCGCACTGGTC-3'
		RET-19	5'-GCTGCTGCTGGCAGAGACCA-3'
**FRA3B**	1	FRA3B-20	5’- BBCCTATCTGACGACTTCAC-3’
		FRA3B-9	5’- GAAAGCATAAAGTGTGGC-3’
		FRA3B-23	5’- TAACTGCTTATTTTTCCGATGT-3’
**12p12.3**	1	12p12.3-1	5’-BBTTTTCTTGACTAGTCTAACCAGAT-3’
		12p12.3-2	5’-TTTCACTTGTATTGATCTCCTTCAT-3’
		12p12.3-3	5’-TTTCCACTGTTTGCCGCATTAT-3’
**Linker**		LL3	5’-CGAGTTCAGTCCGTAGACCATGGAGATCTGAATTC-3’
		LP2	5’-GAATTCAGATCTCC-3’
		LL4	5’-CGAGTTCAGTCCGTAGAC-3’
		LL2	5’-GTAGACCATGGAGATCTGAAATTC-3’

[a]B, biotin
