# Supplementary material for: DNA Topoisomerases Participate in Fragility of the Oncogene RET
Source: PLoS One. 2013 Sep 11;8(9):e75741. doi: 10.1371/journal.pone.0075741 (PMC3770543; doi:10.1371/journal.pone.0075741)
Supplement: Table S2 — APH‐Induced DNA breakpoints within RET Intron 11. (PDF) [file pone.0075741.s004.pdf]

Table S2. APH-Induced DNA Breakpoints within *RET* Intron 11.

Breakpoint Number	Strand[a]	Distance From Exon 11 (bp)	Distance to Closest Patient Breakpoint (bp)[b]	Distance to Closest Predicted Topo I Cleavage Site (bp)[b]	Distance to Closest Predicted Topo II Cleavage Site (bp)[b]
1	Top	48	-15	-19	+12
2	Bottom	86	+35	+4	-8
3	Bottom	100	+21	-10	+2
4	Bottom	102	+19	-12	0
5	Bottom	116	+5	+8	-3
6	Bottom	150	+18	+3	+9
7	Bottom	152	+16	+1	+7
8	Bottom	154	+14	-1	+5
9	Top	173	-1	-6	-1
10	Bottom	211	+33	-3	+7
11	Bottom	212	+32	-4	+6
12	Bottom	215	+29	+4	+3
13	Bottom	215	+29	+4	+3
14	Top	233	+11	+2	-5
15	Bottom	238	+6	+6	+5
16	Bottom	249	-2	+3	-6
17	Bottom	277	-24	-3	-9
18	Bottom	280	-27	+5	+9
19	Bottom	283	-30	+2	+6
20	Bottom	294	-41	+4	+3
21	Bottom	297	-44	+1	0
22	Bottom	297	-44	+1	0
23	Bottom	326	+29	-5	0
24	Bottom	329	+26	+2	-3
25	Bottom	348	+7	+1	-2
26	Bottom	356	+1	+3	+5
27	Top	378	-7	-6	+3
28	Top	440	0	0	+1
29	Bottom	466	-1	-3	-8
30	Top	500	+6	+7	-1
31	Bottom	502	+4	-1	+1
32	Bottom	507	-1	+1	0
33	Bottom	556	+31	-5	-3
34	Top	584	+3	-7	+2
35	Bottom	592	-1	+8	-2
36	Top	605	-7	-2	-3
37	Top	651	-53	+1	-8
38	Bottom	680	+57	+6	-1
39	Bottom	687	+50	-1	+6
40	Bottom	694	+43	0	0

41	Top	706	+31	+1	-1
42	Top	724	+13	-17	+3
43	Bottom	735	+2	-8	-4
44	Bottom	755	-1	-7	0
45	Top	794	-40	-1	0
46	Top	806	+34	+11	+2
47	Top	806	+34	+11	+2
48	Top	806	+34	+11	+2
49	Top	806	+34	+11	+2
50	Top	806	+34	+11	+2
51	Top	806	+34	+11	+2
52	Top	806	+34	+11	+2
53	Top	806	+34	+11	+2
54	Top	809	+31	+8	-1
55	Top	809	+31	+8	-1
56	Top	815	+25	+2	0
57	Bottom	830	10	+9	0
58	Top	845	0	-4	0
59	Bottom	845	0	-4	0
60	Top	855	-10	0	+4
61	Top	855	-10	0	+4
62	Top	898	-6	+5	-1
63	Top	900	-8	+3	-3
64	Top	935	+9	-6	+1
65	Bottom	941	+3	-3	-1
66	Top	945	0	+3	+3
67	Bottom	970	-12	-4	+3
68	Top	986	+28	0	0
69	Top	993	+21	0	-2
70	Top	996	+18	-1	+2
71	Bottom	999	+15	-4	-8
72	Bottom	1028	+9	-5	-1
73	Bottom	1036	+2	0	+6
74	Bottom	1045	+4	+3	-3
75	Bottom	1048	+1	0	+2
76	Bottom	1049	0	-1	+1
77	Bottom	1094	+2	0	+3
78	Bottom	1142	-5	+1	+1
79	Bottom	1142	-5	+1	+1
80	Bottom	1182	-45	-1	+4
81	Bottom	1186	-49	+1	0
82	Top	1210	+45	-4	-2
83	Top	1214	+41	+7	+2
84	Bottom	1222	+33	-1	-2

85	Bottom	1223	+32	-2	-3
86	Top	1239	+16	+3	+3
87	Bottom	1241	+14	-1	-2
88	Bottom	1246	+9	-6	0
89	Bottom	1246	+9	-6	0
90	Top	1254	+1	0	-2
91	Top	1260	0	-6	+6
92	Bottom	1269	-5	0	+1
93	Bottom	1282	-3	-7	-3
94	Bottom	1292	-10	+6	-1
95	Bottom	1293	-11	+5	-2
96	Bottom	1309	+24	+1	-1
97	Bottom	1320	+13	0	+6
98	Bottom	1330	-3	-5	-4
99	Top	1332	+1	-9	+6
100	Top	1334	0	-11	+4
101	Bottom	1339	-5	-1	-5
102	Top	1346	-12	0	0
103	Top	1365	-31	-12	-2
104	Bottom	1383	-49	+6	-10
105	Bottom	1391	+58	0	+5
106	Bottom	1394	+55	-3	+2
107	Bottom	1396	+53	+1	0
108	Bottom	1409	+20	-8	+5
109	Top	1419	+30	+18	-5
110	Bottom	1444	+5	+9	-2
111	Top	1453	0	+3	-2
112	Bottom	1456	-3	-3	-1
113	Bottom	1456	-3	-3	-1
114	Top	1462	-9	-4	0
115	Top	1465	-12	-7	-3
116	Top	1474	+21	-1	-4
117	Bottom	1488	+7	-2	0
118	Top	1511	-15	+1	-3
119	Top	1516	-20	-4	+7
120	Top	1519	-23	+4	+4
121	Bottom	1534	+20	-7	0
122	Top	1545	+9	+5	0
123	Top	1556	-2	+2	-3
124	Top	1576	-8	-4	-1
125	Top	1579	-11	+2	+2
126	Top	1581	-13	0	0
127	Top	1581	-13	0	0
128	Top	1583	-15	-2	-2

129	Top	1651	-6	-4	-2
130	Top	1669	+12	+10	+2
131	Bottom	1678	+3	-2	+3
132	Top	1707	+10	+10	-3
133	Top	1708	+9	+9	-4
134	Top	1718	-2	-1	+3
135	Top	1718	-2	-1	+3
136	Top	1720	-3	+1	+1
137	Top	1738	-13	+2	-2
138	Top	1763	+26	-10	-3
139	Bottom	1771	+18	+1	-7
140	Bottom	1772	+17	0	+6
141	Top	1781	+8	+4	-3
142	Top	1785	-4	0	+1
143	Top	1795	0	+1	+4
144	Top	1805	+10	-1	-5

[a] The top strand indicates the strand for which the sequence is shown in Figure 2, and the bottom strand indicates the complementary strand.

[b]A positive distance refers to the patient/topoisomerase cleavage site being downstream from the APH-induced breakpoint, while a negative distance is being upstream.
