# Supplementary material for: DNA Topoisomerases Participate in Fragility of the Oncogene RET
Source: PLoS One. 2013 Sep 11;8(9):e75741. doi: 10.1371/journal.pone.0075741 (PMC3770543; doi:10.1371/journal.pone.0075741)
Supplement: Table S3 — Location of RET Intron 11 APH‐Induced DNA breakpoints on predicted DNA secondary structures. (PDF) [file pone.0075741.s005.pdf]

Table S3. Location of *RET* Intron 11 APH-Induced DNA Breakpoints on Predicted DNA Secondary Structures.

Breakpoint Number	Strand[a]	Distance From Exon 11 (bp)	Double-Stranded/ Single-Stranded DNA Junction[b]	Double-Stranded DNA Stem	Single-Stranded DNA Loop	Single-Stranded DNA Bubble
1	Top	48		x		
2	Bottom	86	x			
3	Bottom	100		x		
4	Bottom	102		x		
5	Bottom	116	x			
6	Bottom	150	x			
7	Bottom	152		x		
8	Bottom	154		x		
9	Top	173	x			
10	Bottom	211				x
11	Bottom	212				x
12	Bottom	215		x		
13	Bottom	215		x		
14	Top	233	x			
15	Bottom	238		x		
16	Bottom	249		x		
17	Bottom	277	x			
18	Bottom	280		x		
19	Bottom	283		x		
20	Bottom	294		x		
21	Bottom	297	x			
22	Bottom	297	x			
23	Bottom	326	x			
24	Bottom	329				x
25	Bottom	348			x	
26	Bottom	356	x			
27	Top	378		x		
28	Top	440	x			
29	Bottom	466				x
30	Top	500	x			
31	Bottom	502	x			
32	Bottom	507	x			
33	Bottom	556		x		
34	Top	584		x		
35	Bottom	592		x		
36	Top	605			x	
37	Top	651		x		
38	Bottom	680		x		

39	Bottom	687		x		
40	Bottom	694		x		
41	Top	706		x		
42	Top	724		x		
43	Bottom	735	x			
44	Bottom	755		x		
45	Top	794	x			
46	Top	806		x		
47	Top	806		x		
48	Top	806		x		
49	Top	806		x		
50	Top	806		x		
51	Top	806		x		
52	Top	806		x		
53	Top	806		x		
55	Top	809		x		
54	Top	809	x			
56	Top	815	x			
57	Bottom	830	x			
59	Bottom	845		x		
58	Top	845	x			
60	Top	855		x		
61	Top	855		x		
62	Top	898		x		
63	Top	900	x			
64	Top	935			x	
65	Bottom	941		x		
66	Top	945		x		
67	Bottom	970		x		
68	Top	986	x			
69	Top	993		x		
70	Top	996		x		
71	Bottom	999	x			
72	Bottom	1028			x	
73	Bottom	1036	x			
74	Bottom	1045			x	
75	Bottom	1048				x
76	Bottom	1049	x			
77	Bottom	1094	x			
78	Bottom	1142		x		
79	Bottom	1142		x		
80	Bottom	1182			x	
81	Bottom	1186			x	
82	Top	1210		x		

83	Top	1214				x
84	Bottom	1222	x			
85	Bottom	1223		x		
86	Top	1239				x
87	Bottom	1241	x			
88	Bottom	1246	x			
89	Bottom	1246	x			
90	Top	1254			x	
91	Top	1260		x		
92	Bottom	1269		x		
93	Bottom	1282	x			
94	Bottom	1292				x
95	Bottom	1293				x
96	Bottom	1309		x		
97	Bottom	1320				x
98	Bottom	1330			x	
99	Top	1332			x	
100	Top	1334			x	
101	Bottom	1339	x			
102	Top	1346			x	
103	Top	1365	x			
104	Bottom	1383	x			
105	Bottom	1391				x
106	Bottom	1394	x			
107	Bottom	1396			x	
108	Bottom	1409	x			
109	Top	1419		x		
110	Bottom	1444	x			
111	Top	1453	x			
112	Bottom	1456			x	
113	Bottom	1456			x	
114	Top	1462			x	
115	Top	1465		x		
116	Top	1474			x	
117	Bottom	1488				x
118	Top	1511		x		
119	Top	1516		x		
120	Top	1519			x	
121	Bottom	1534		x		
122	Top	1545	x			
123	Top	1556			x	
124	Top	1576	x			
125	Top	1579	x			
126	Top	1581		x		

127	Top	1581		x		
128	Top	1583		x		
129	Top	1651		x		
130	Top	1669		x		
131	Bottom	1678		x		
132	Top	1707			x	
133	Top	1708			x	
134	Top	1718	x			
135	Top	1718	x			
136	Top	1720	x			
137	Top	1738	x			
138	Top	1763	x			
139	Bottom	1771		x		
140	Bottom	1772			x	
141	Top	1781	x			
142	Top	1785	x			
143	Top	1795	x			
144	Top	1805		x		

[a]The top strand indicates the strand for which the sequence is shown in Figure 2, and the bottom strand indicates the complementary strand.

[b]A breakpoint is located within the double-stranded DNA stem, one nucleotide from the base of the stem with allowance of up to a 5-nt deletion. The allowances for deletion is based upon the observation of up to a 5-nt deletion of BanI-induced DNA breakage within *RET* intron 11 as described in the results.
